# Desmoglein‐3 induces YAP phosphorylation and inactivation during collective migration of oral carcinoma cells

**DOI:** 10.1002/1878-0261.13177

**Published:** 2022-03-01

**Authors:** Usama Sharif Ahmad, Eric Kenneth Parkinson, Hong Wan

**Affiliations:** ^1^ Centre for Oral Immunobiology and Regenerative Medicine Institute of Dentistry Barts and The London, School of Medicine and Dentistry UK

**Keywords:** collective cell migration, desmoglein‐3, Hippo pathway, oral carcinoma cell line, phosphorylated YAP, YAP

## Abstract

Alterations of the Hippo–YAP pathway are potential targets for oral squamous cell carcinoma (OSCC) therapy, but heterogeneity in this pathway could be responsible for therapeutic resistance. We analysed the Hippo–YAP signatures in a cohort of characterised keratinocyte cell lines derived from the mouth floor and buccal mucosa from different stages of OSCC tumour progression and focused on the specific role of YAP on invasive and metastatic potential. We confirmed heterogeneity in the Hippo–YAP pathway in OSCC lines, including overexpression of *YAP1*, *WWTR1* (often referred to as TAZ) and the major Hippo signalling components, as well as the variations in the genes encoding the intercellular anchoring junctional proteins, which could potentially regulate the Hippo pathway. Specifically, desmoglein‐3 (DSG3) exhibited a unique and mutually exclusive regulation of YAP via YAP phosphorylation during the collective migration of OSCC cells. Mechanistically, such regulation was associated with inhibition of phosphorylation of epidermal growth factor receptor (EGFR) (S695/Y1086) and its downstream effectors heat shock protein beta‐1 (Hsp27) (S78/S82) and transcription factor AP‐1 (c‐Jun) (S63), leading to YAP phosphorylation coupled with its cytoplasmic translocation and inactivation. Additionally, OSCC lines displayed distinct phenotypes of YAP dependency or a mixed YAP and TAZ dependency for cell migration and present distinct patterns in YAP abundance and activity, with the latter being associated with YAP nuclear localisation. In conclusion, this study provides evidence for a newly identified paradigm in the Hippo–YAP pathway and suggests a new regulation mechanism involved in the control of collective migration in OSCC cells.

## Graphical abstract

Here, we investigated the reciprocal regulation between DSG3 and YAP in oral squamous cell carcinoma (OSCC) cells. We observed that low DSG3 expression was coupled with activation of YAP, which was predominantly located in the nucleus, via EGFR signalling. In contrast, in cells expressing high levels of DSG3, YAP was phosphorylated and translocated to the cytoplasm, via inhibition of EGFR signalling. Altogether, high DSG3 levels lead to contact inhibition of cell locomotion, and attenuated collective cell migration of OSCC cells.

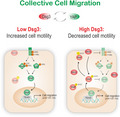

**Abbreviations**
AJ: adherens junctions
ANOVA: analysis of variance
BM: buccal mucosa
BPE: bovine pituitary extract
cDNA: complementary deoxyribonucleic acid
c‐Jun: transcription factor AP‐1
Cyto: cytoplasmic
DAPI: 4′,6‐diamidino‐2‐phenylindole
DMEM: Dulbecco's modified Eagle medium
DNA: deoxyribonucleic acid
Dp: Desmoplakin
DSG2: desmoglein‐2
DSG3: desmoglein‐3
DSM: desmosome
E‐cad: E‐cadherin
EDTA: ethylenediaminetetraacetic acid
EGFR: epidermal growth factor receptor
EMT: epithelial‐to‐mesenchymal transition
F: fibroblast‐like
FA: focal adhesions
FAK: focal adhesion kinase
FBS: foetal bovine serum
FOM: floor of mouth
FOXM1: Forkhead box protein M1
GAPDH: glyceraldehyde‐3‐phosphate dehydrogenase
HBSS: Hanks' balanced salt solution
HC: highly compact
HNSCC: head and neck squamous cell carcinoma
Hsp27: heat shock protein beta‐1
IgG: immunoglobulin G
IMF: immunofluorescence
K14: keratin 14
KD: knockdown
KSFM: keratinocyte‐serum‐free medium
LATS1/2: large tumour suppressor 1 and 2 kinases
LC: loosely associated/compact
Luci: luciferase
MAPK: mitogen‐activated protein kinase
MC: medium compact
MIQE: Minimum Information for Publication of Quantitative Real‐Time PCR Experiments
MMC: mitomycin C
MOB1: MOB kinase activator 1
mRNA: messenger ribonucleic acid
MST1/2: mammalian Sterile 20‐related 1 and 2 kinases
Nuc: nuclear
OC: oral cancer
OSCC: oral squamous cell carcinoma
PAGE: polyacrylamide gel electrophoresis
PBS: phosphate‐buffered saline
PCNA: proliferating cell nuclear antigen
PCR: polymerase chain reaction
p‐FAK: phosphorylated‐focal adhesion kinase, Tyr397/Y397
Pg: plakoglobin
PKP1: plakophilin‐1
PKP3: plakophilin‐3
p‐YAP: phosphorylated yes‐associated protein S127
qPCR: quantitative polymerase chain reaction
rEGF: recombinant epidermal growth factor
RNAi: ribonucleic acid interference
RT: room temperature
RT‐qPCR: quantitative reverse transcription polymerase chain reaction
SAV1: Salvador 1
SCC: squamous cell carcinoma
Scram: scrambled
SD: standard deviation
SDS: sodium dodecyl sulphate
SEM: standard error of the mean
siRNA: small interfering ribonucleic acid
STAT3: signal transducer and activator of transcription 3
TAZ: transcriptional coactivator with PDZ‐binding motif
TEAD: transcriptional enhanced associate domain
TTBS: tris‐buffered saline containing 0.1% Tween 20
UVB: ultraviolet B
V: voltage
YAP: yes‐associated protein
α‐Cat: α‐Catenin
β‐Cat: β‐Catenin

## 1 Introduction

Head and neck squamous cell carcinoma (HNSCC) is the 8th most common cancer in the UK, with incidence rates increasing by a fifth (20%) over the last decade. Worldwide, HNSCC accounted for more than 800 000 new cases with 450 000 deaths in 2018, among which oral squamous cell carcinoma (OSCC) accounted for 350 000 new cases and 170 000 deaths [1]. OSCC can develop from recognisable premalignant lesions, but this is not always the case [1]. Patients with advanced disease in this type of cancer often suffer from substantial functional and cosmetic defects after surgery which severely affects the quality of patient life. The rapid advance in comprehensive ‘multi‐omics’ signature and signalling pathway studies has highlighted the heterogeneity and the most common oncogenic driver mutations as well as the pathways that promote progression in OSCC [1]. Tumour heterogeneity is thought to attribute to the resistance to treatment and failure. The study of cancer cell lines offers the advantage to explore the diversity of molecular and pathogenic alterations, such as mutations, copy number variations, gene and protein expression profiles, that featuring each cell line without any influence by the complexities of whole tissues [2, 3, 4, 5].

Recent studies have identified genetic alterations of the Hippo pathway in HNSCC [6]. This evolutionarily conserved Hippo pathway is crucial to control tissue homeostasis and tumorigenesis by inhibiting two paralogous transcription cofactors Yes‐associated protein (YAP) and an effector with PDZ‐binding motif (TAZ). Both YAP/TAZ shuttle between the nucleus and cytoplasm depending on multiple upstream inputs that regulate Hippo signalling. The Hippo pathway comprises a cascade of serine/threonine kinases, including MST1/2 (STK4/3) and its associated adaptor protein SAV1, their targets LATS1/2 kinases and an adaptor protein MOB1 [6, 7, 8]. When Hippo signalling is on (active), YAP/TAZ are subject to phosphorylation by LATS1/2, leading to their nuclear exclusion and cytoplasm retention via binding to 14‐3‐3 protein or ubiquitination‐mediated degradation. When Hippo is off, the active YAP/TAZ translocate to the nucleus where they interact with the DNA‐binding protein TEAD transcription factors that are responsible for most proliferative, anti‐apoptotic and oncogenic activities of YAP/TAZ [9, 10]. Thus, it is not surprising that both YAP and TAZ are overexpressed in various cancers, including those of the head and neck [6, 8, 11, 12]. Emerging evidence suggests that YAP and TAZ possess distinct nonoverlapping transcription programs in cancers [5, 13]. While YAP preferentially regulates genes associated with cell proliferation, TAZ favourably governs genes associated with extracellular matrix and cell motility [13]. A study based on tissue microarray sections of 166 tumours found increased YAP in 24% of patients with high nuclear YAP correlated with poorer overall survival [3]. Dysregulated signalling by the Hippo‐YAP pathway in cancer is appreciated but the molecular drivers and regulators that affect Hippo signalling in cancer development have not been fully understood.

Desmoglein‐3 (DSG3), an isoform of the desmoglein subfamily, is characterised as the primary autoantigen in pemphigus vulgaris, an autoimmune blistering disease of the skin and oral mucous membrane [14]. The circulating autoantibodies targeting DSG3 cause disruption of cell cohesion and the loss of DSG3 from the keratinocyte surface. DSG3 expression is not solely restricted to desmosomes but rather presents on the entire keratinocyte surfaces. The function of nonjunctional DSG3 remains not fully understood; however, it was thought that it is associated with cell signalling and is the primary target of pemphigus IgG [15]. Although pemphigus is a rare condition, there are still a few case reports in the literature that show OSCC arising in the oral cavity of patients with pemphigus and present with delayed cervical lymph node metastasis, suggesting a likelihood of potential link between pemphigus and malignancy [16, 17, 18]. Our recent study has shown that desmoglein‐3 (DSG3) can regulate YAP in keratinocytes by complexing with its phosphorylated form and recruiting it preferentially to the cell surface [19]. Intriguingly, DSG3 silencing resulted in a reduction of YAP and *YAP1* target genes that are associated with cell proliferation, suggesting that DSG3 somehow is capable of influencing YAP nuclear activity [20]. Concordantly, upregulation of DSG3 is found in cancers of various organs, including the head and neck [21]. In support, ectopic expression of DSG3 in the A431 carcinoma cell line elicits activation of several signalling pathways that lead to enhanced cell migration and invasion [20, 22, 23, 24, 25]. However, YAP can also negatively regulate DSG3 [26]. Thus, the relationship between DSG3 and YAP remains unclear. In this present study, we sought to identify the molecular signatures of the Hippo‐YAP pathway in a panel of authenticated oral cell lines of normal, dysplasia and carcinoma derived from buccal mucosa (BM) and floor of mouth (FOM) and then focused on the role of YAP in the metastatic potential of OSCC cells. Our findings from this study highlight the heterogeneous nature of the Hippo‐YAP pathway in OSCC cell lines and provide evidence for partially overlapping activities of YAP and TAZ in the control of cell migration. Additionally, we show that the regulation of DSG3 and YAP in OSCC cells is mutually exclusive.

## 2 Materials and methods

### 2.1 Cell lines and culture conditions

Normal immortalised human oral keratinocyte lines OKF6/TERT‐1 (OKF6), OKF4/CDK4R/P53DD/TERT (OKF4) [27] and dysplasia oral keratinocyte D4 [28, 29] were grown in keratinocyte‐serum‐free medium (KSFM) (Thermo Fisher Scientific, Dartford, UK) supplemented with 0.2 ng·mL^−1^ human recombinant epidermal growth factor (rEGF) and 12.5 µg·mL^−1^ bovine pituitary extract (BPE). Normal oral keratinocyte line SLC002 [30] and dysplasia cell line D17‐TERT [29, 31] were grown in basal medium, that is Dulbecco's modified Eagle medium (DMEM, Lonza, Basel, Switzerland)/Ham's F‐12 (Thermo Fisher Scientific) 1 : 1 supplemented with 10% (v/v) foetal bovine serum (FBS; Thermo Fisher Scientific) and 0.5 µg·mL^−1^ hydrocortisone (Sigma, Dorset, UK) in the presence of lethally irradiated Swiss 3T3 fibroblasts [32]. Cells were incubated at 37 °C in a humidified atmosphere of 5% CO_2_/95% air, changing medium every 1–2 days. Human OSCC cell lines H376, H314, H157 and H413 [33] all were also cultured in basal medium, and SqCC/Y1 line [34] was cultured in KSFM. Cells were incubated at 37 °C in a humidified atmosphere of 5% CO_2_/95% air, changing medium every 1–2 days. For all experiments, all cell lines were seeded and cultured in basal medium.

The OKF6/TERT‐1 (OKF6) [27] and OKF4/CDK4R/P53DD/TERT (OKF4) [35] lines were a generous gift from J. G. Rheinwald. The H157, H314, H376 and H413 cell lines were a generous gift from S. Prime [36], and the details of D4 [29] and D17‐TERT [37] lines were published previously. The SqCC/Y1 line was from the centre and published previously [22]. The normal keratinocyte line SLC002 was derived from the buccal mucosa of a control subject with informed consent (Dental Teaching Hospital, Peradeniya, Sri Lanka, and Ethical Clearance Certificate No. FDS‐ERC/2008/02/TIL) [30].

### 2.2 Antibodies

The following mouse and rabbit monoclonal/polyclonal antibodies (Abs) were used: D8H1X, rabbit Ab to YAP (14074; Cell Signalling Technology, Leiden, Netherlands); EP1675Y, rabbit Ab to YAP1 (phospho S127) (ab76252; Abcam, Cambridge, UK); D24E4, rabbit Ab to YAP/TAZ (8418; Cell Signalling Technology); LATS1/2, rabbit Ab (9153; Cell Signalling Technology); 5H10, mouse Ab against the N‐terminus of Dsg3 (sc‐23912; Santa Cruz, Dallas, TX, USA); 33–3D, mouse IgM against Dsg2 (gift from Professor Garrod); PG 5.1, mouse Ab to Plakoglobin (65015; Progen, Heidelberg, Germany); H‐300, rabbit Ab to Desmoplakin (sc‐33555; Santa Cruz); 5C2, mouse Ab to PKP1 (Progen); PKP3, mouse Ab (ab151401; Abcam); HECD‐1, mouse anti‐N terminus of E‐Cadherin (ab1416; Abcam); rabbit Ab to α‐Catenin (C2081; Sigma); 6F9, mouse Ab to β‐Catenin (C7082; Sigma); mouse Ab to K14 (gift from Professor Leigh); C‐20, rabbit Ab to FAK (sc‐558; Santa Cruz); Anti‐phospho‐FAK (pTyr^397^) rabbit Ab (SAB4504403; Sigma); D38B1, rabbit Ab to EGF Receptor (4267; Cell Signalling Technology); D7A5, rabbit Ab to Phospho‐EGF Receptor (Y1068) (3777; Cell Signalling Technology); Phospho‐EGFR (S695), rabbit Ab (PA5‐104725; Thermo Fisher Scientific); Phospho‐EGFR (Y1086), rat Ab (MAB89671; R&D Systems, Minneapolis, MN, USA); 60A8, rabbit Ab to c‐Jun (9165; Cell Signalling Technology); 54B3, rabbit Ab to Phospho‐c‐Jun (S63) (2361; Cell Signalling Technology); G31, mouse Ab to HSP27 (2402; Cell Signalling Technology); Phospho‐HSP27 (S78), rabbit Ab (2405; Cell Signalling Technology); Phospho‐HSP27 (S82), rabbit Ab (2401; Cell Signalling Technology); Alexa Fluor 488 conjugated phalloidin for F‐Actin (A12379; Thermo Fisher Scientific); C‐20, rabbit Ab to FOXM1 (sc‐502; Santa Cruz); H‐432, rabbit Ab to Cyclin A (sc‐751; Santa Cruz); PC10, mouse Ab to PCNA (sc‐56; Santa Cruz); Y69, rabbit Ab to c‐Myc (ab32072; Abcam); MIB‐1, mouse Ab to Ki‐67 (GA626; Agilent, Santa Clara, CA, USA); 14C10, rabbit Ab to GAPDH (2118; Cell Signalling Technology); B‐6, mouse Ab to HSC 70 (sc‐7298; Santa Cruz); Secondary Abs were anti‐mouse/rabbit IgG peroxidase antibody produced in goat (A0168/A6667; Sigma); Alexa Fluor 488 goat anti‐mouse/rabbit IgG (A11029/A11034; Thermo Fisher Scientific), and Alexa Fluor 568 goat anti‐mouse/rabbit IgG (A11031/A11036; Thermo Fisher Scientific).

### 2.3 siRNA transfection

Transient YAP siRNA transfection was performed in H157 and H413 cells as described previously [19, 38]. Two siRNA sequences were purchased from Dharmacon™ (Lafayette, CO, USA) ON‐TARGETplus siRNA‐7J‐012200‐07 and siRNA‐8J‐012200‐08, Human *YAP1* NM‐001130145 (siRNA‐7: GGTCAGAGATACTTCTTAA referred as siRNA‐1 and siRNA‐8: CCACCAAGCTAGATAAAGA referred as siRNA‐2), along with the scrambled control provided by the same company. A custom siRNA sequence specific for human *WWTR1* (ACGUUGACUUAGGAACUUU) [39] was synthesised by Dharmacon™. In brief, 2 × 10^6^ cells were seeded in a 100 mm dish overnight before transfected with either scrambled or specific siRNA at a final concentration of 50 nm using DharmaFECT 1 (Dharmacon) following the manufacturer’s instructions. The next day, cells were harvested with 0.25% Trypsin/EDTA and re‐plated for various assays or analyses by different techniques as described previously [19, 38].

### 2.4 Generation of H413 stable line with transduction of full‐length Dsg3

The retroviral constructs of full‐length human Dsg3 cDNA (pBABE‐hDsg3.myc), tagged with a Myc epitope at C terminus, was generated before in this laboratory [20]. The method used to generate stable H413 cell line with transduction of pBABE‐hDsg3.myc or empty vector control was described previously [20].

### 2.5 Quantitative reverse transcription polymerase chain reaction (RT‐qPCR)

The method was described in a previous study [19]. Briefly, mRNA extraction was conducted using the Dynabeads*™* mRNA DIRECT™ Purification Kit (Thermo Fisher Scientific) and was converted to cDNA using the qPCRBIO cDNA Synthesis Kit (PCR Biosystems, London, UK) and cDNA was diluted 1 : 4 with RNase/DNase‐free water and stored at −20 °C until required for qPCR. RT‐qPCR was performed using the qPCRBIO SyGreen Blue Mix Lo‐ROX (PCR Biosystems) in the LightCycler^®^ 480 Multiwell Plate 384 (Roche Life Science, Basel, Switzerland) according to our well‐established protocols [40] which are Minimum Information for Publication of Quantitative Real‐Time PCR Experiments (MIQE) compliant [41]. Briefly, thermocycling begins with 95 °C for 30 s prior to 45 cycles of amplification at 95 °C for 1 s, 60 °C for 1 s, 72 °C for 6 s, 76 °C for 1 s (data acquisition). A ‘touch‐down’ annealing temperature intervention (66 °C starting temperature with a stepwise reduction of 0.6 °C/cycle; 8 cycles) was introduced prior the amplification step to maximise primer specificity. Melting analysis (95 °C for 30s, 65 °C for 30 s, 65–99 °C at a ramp rate of 0.11 °C·s^−1^) was performed at the end of qPCR amplification to validate single product amplification in each well. The advanced relative quantification of mRNA expression was calculated based on an objective method using the second derivative maximum algorithm using the LightCycler^®^ 480 Software (Roche Life Science). *B2M* and *POLR2A* were used as stable reference genes to normalise all target genes. Each sample was run in duplicates or quadruplicates. The primer sequences for all genes analysed in this study are listed in Table S3.

### 2.6 Cell migration assay

Cell migration was evaluated using the Oris™ Cell Migration Assay Kit (PlatyPus Technologies, Oxford, UK) according to the manufacturer’s instructions. In brief, siRNA‐transfected H157 and H413 cells were first treated with 5 μm CellTracker™ Deep Red (Thermo Fisher Scientific) in PBS and incubated at 37 °C for 30 min and then seeded (7.5 × 10^4^ per well) into the wells of the Oris™ 96‐well plate containing Oris™ cell seeding stoppers in quadruplicate. After 24 h, cells were treated with 10 μg·mL^−1^ Mitomycin C (Sigma) for 3 h before allowing the cells to migrate into the detection zone after removal of the stoppers. Image acquisition of premigration and postmigration were taken with a 4× objective using INCA 2200 Imaging System (GE Healthcare, Chicago, IL, USA). Images were analysed using the in cell developer toolbox Software (GE Healthcare).

Additionally, time‐lapse microscopy was performed in sparse cultures of YAP siRNA‐treated cells as well as the H413 Vect Ct and hDsg3.myc cell lines in 6‐well plate with a 10× objective using INCA 2200 Imaging System at an interval of 20 min for 16–24 h. Videos and manual cell tracking were made with ImageJ and graphs were generated with the ibidi Chemotaxis and Migration tool. The accumulated distance and velocity of random cell migration were analysed in an Excel spreadsheet.

### 2.7 Western blotting analysis

This method was also described in our previous studies [20, 23]. Briefly, cell extraction was performed to isolate proteins from cultures at approximately 70–80% confluence. Cells were washed with PBS and extracted with 2× sodium dodecyl sulphate (SDS) Laemmli sample buffer (0.5 m Tris‐Cl pH 6.8, 4% SDS, 20% Glycerol; 10% (v/v) 2‐mercaptoethanol was added after protein assay) immediately. DC Protein Assay (Bio‐Rad, Hercules, CA, USA) was used to determine the protein concentration for each sample. Equal loading was confirmed before probing for the target proteins in each set of samples. 10–40 μg of total protein was resolved by SDS/PAGE at 80–120 V and then transferred to a nitrocellulose membrane (cytiva) at 30 V in an electrophoresis apparatus. The nonspecific binding sites on the membrane were blocked for 30 min at room temperature (RT) in blocking buffer (5% (w/v) nonfat dry milk in TTBS (Tris buffer containing 0.1% Tween 20)), and then, the membrane was incubated with primary antibody against specific proteins at appropriate dilutions for each antibody, for overnight at 4 °C. After three washes in TTBS, the membrane was incubated with the secondary antibody conjugated with HRP (rabbit‐SAB3700846, mouse‐A0168; Sigma) at appropriate dilutions in blocking buffer for 1 h at RT. Following three washes in TTBS, the membrane was subjected to Pierce™ ECL Western Blotting Substrate (Thermo Fisher Scientific) following the manufacturer’s instructions. The membrane was then exposed to Amersham Hyperfilm ECL (VWR, Leicestershire, UK) and developed in an AGFA Curix 60 Developer (Blizard Institute Core Facility) in a dark room to detect target proteins.

### 2.8 Immunofluorescence, microscopy and image analysis

As described in our previous studies [19, 38], cultured cells grown on coverslips were fixed in 3.6% formaldehyde in PBS for 10 min and permeabilised with 0.1% Triton X‐100 for 5 min. The nonspecific binding sites were blocked with 10% goat serum (Sigma) for 15–30 min before primary and secondary antibody incubations, each for 1 h, at room temperature, respectively. All antibodies were diluted in 10% goat serum. Coverslips were washed three times with washing buffer (PBS containing 0.2% Tween 20) after each antibody incubation. Finally, coverslips were counterstained with DAPI for 8–10 min and were mounted on slides. For image quantitation, all images were routinely acquired in 4–6 arbitrary fields per sample using Leica DM4000 Epi‐Fluorescence Microscope or Zeiss 710 Laser Scanning Confocal Microscope (Blizard Institute Core Facility). All images were analysed with fiji Software (version 1.53) [3]. For analysis of cytoplasmic and nuclear immunofluorescence (IMF) signals, each image was measured before and after being subtracted by the binary image of the DAPI channel for total and cytoplasmic signals, respectively. The nuclear signals were calculated by subtracting the cytoplasmic signals from the total IMF value for each image in an Excel spreadsheet. Finally, the mean IMF intensity per cell for each image was calculated by dividing the total or cytoplasm/nuclear signals, with the cell number before statistical analysis. Data were presented as the mean IMF intensities for total, the cytoplasmic and nuclear ratio in each condition are shown in the graphs. For the analysis of FAK and p‐FAK expression and morphological changes in focal adhesions (FA), confocal image stacks focused on the membrane projections were acquired. Then, image stacks were subjected to Z projection with the maximum intensity with fiji imagej before a threshold was chosen. Finally, the Feret's diameter, area, circularity and mean grey value were selected and analysed.

### 2.9 Luciferase assay

YAP/TAZ luciferase assay was performed as described in our previous report [38], using the Bio‐Glo™ Luciferase Assay System (Promega, Southampton, UK) according to the manufacturer’s instructions. Briefly, H157 and H413 cells were seeded at 1 × 10^5^ in a 24‐well plate overnight before transfection with 0.25 μg YAP/TAZ luciferase reporter plasmid (Plasmid #34615; Addgene, Watertown, MA, USA) per well using FuGENE^®^ HD Transfection Reagent (Promega). After 24 h, cells were washed twice with PBS before starting the luciferase assay.

### 2.10 Dispase dissociation assay

The cell–cell adhesion strength was determined by the dispase dissociation assay as described previously [42]. Briefly, cells were seeded at equal confluent densities (2 × 10^6^ cells) in 6‐well plates. Once cells reached confluent monolayers after 2–3 days, cells were washed twice with PBS and then incubated in 2 ml HBSS (Thermo Fisher Scientific) containing dispase II (2.4 U·mL^−1^; Thermo Fisher Scientific) for approximately 15–20 min at 37 °C in 5% CO_2_/95% air until the epithelial sheets began detaching. Next, the epithelial sheets were carefully washed with PBS twice before subjected to mechanical stress by pipetting 2–3 times with a 1 mL pipette to break the epithelial sheets. Fragments in each well were imaged using the ChemiDoc MP Imaging System (Bio‐Rad, Blizard Institute Core Facility) with image lab Software (Bio‐Rad). Finally, the number of fragments for each sample was determined with fiji Software. At least three independent experiments were performed in each cell line, with each experiment carried out in triplicates, and the pooled data were used for statistical analysis.

### 2.11 Human phospho‐kinase profiler array

H413 Vect Ct and hDsg3.myc cell lines seeded at equal density (80–90%) in 10 cm culture dishes were extracted and the phospho‐kinase array was carried out using 500 μg of total protein in each sample, according to the manufacturer’s instructions (ARY003C, Proteome Profiler™ Array; R&D Systems Europe Ltd).

### 2.12 Statistical analysis

For multiple comparisons, the two‐way analysis of variance (ANOVA) was used to determine statistically significant difference. For the comparison of two groups, the unpaired, two‐tailed Student’s *t*‐test was used to obtain *P*‐values. A *P*‐value of less than 0.05 was regarded as statistically significant. All experiments were performed independently for 2–3 times with 2–4 technical replicates.

## 3 Results

### 3.1 YAP overexpression is associated with oral malignancy

**Fig. 1:**
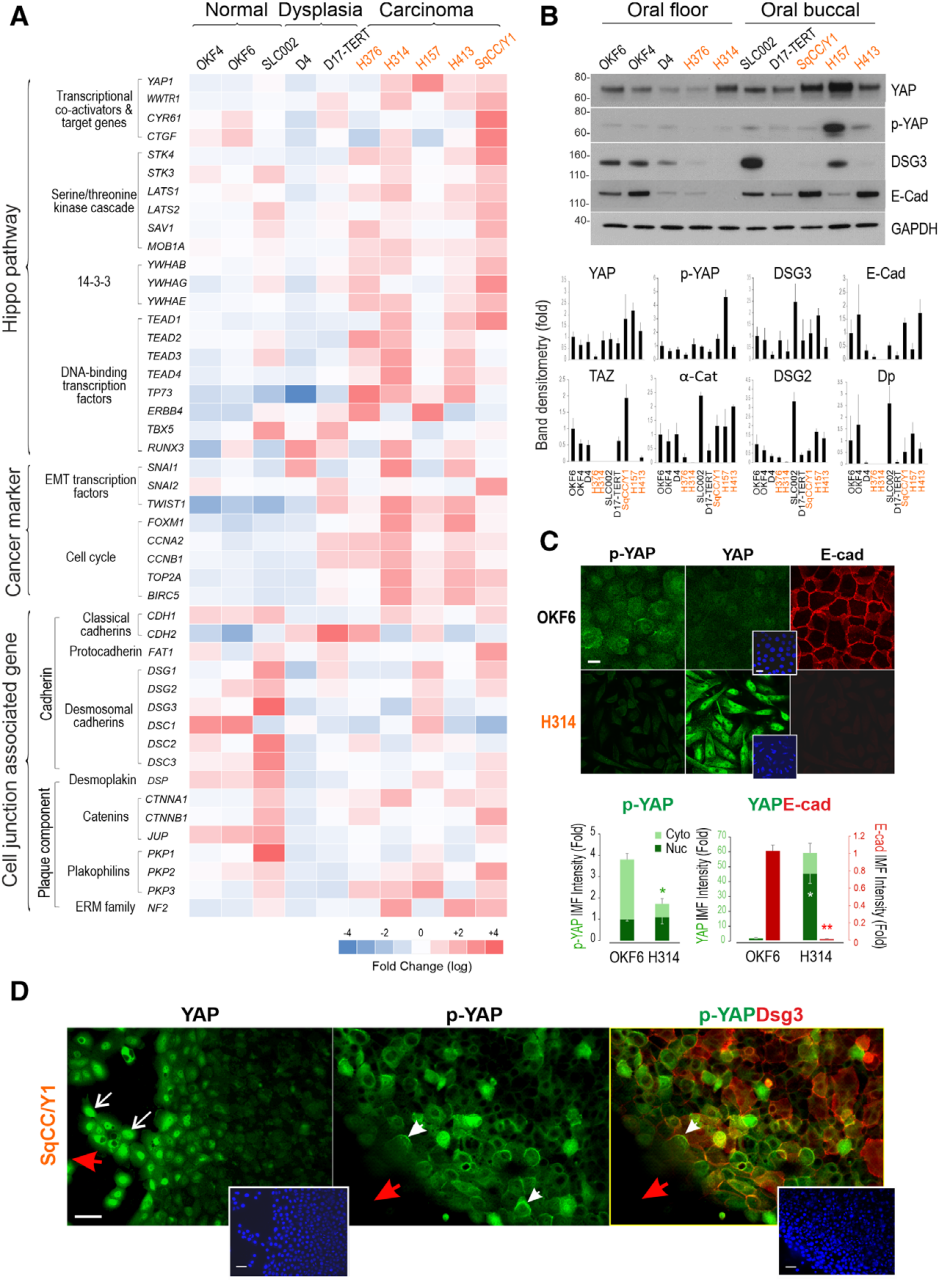
The Hippo‐YAP signatures in a panel of oral keratinocyte cell lines. (A) Heat map of the 46 genes in ten cell lines derived from the oral floor and buccal mucosa according to hierarchical clustering, that is normal, dysplasia and squamous carcinoma cell lines (*x*‐axis). Three categories of the genes were analysed (*y*‐axis), that is Hippo pathway (21 genes), cancer biomarkers (eight genes) and cell junction‐associated genes (17 genes). Data were pooled from two independent biological samples of duplicated qPCR per attempt. Gene expression was normalised against two internal control genes *POLR2A* and *B2M*. The heat map was generated based on the fold change (log_2_) of the mean signal intensity of all ten cell lines. The colour scale reflects the upregulation (red) and downregulation (blue). The carcinoma cell lines are highlighted in orange. (B) Western blotting analysis of total cell lysate for various proteins including YAP in ten oral cell lines (a representative from two independent biological samples). GAPDH was used as a loading control. Cell lines were grouped according to the anatomical site of the oral cavity, that is mouth floor and buccal (mean ± SEM). (C) Confocal images of OKF6 and H314 cell lines for yes‐associated protein (YAP) and phosphorylated YAP (p‐YAP) (green) as well as E‐cadherin (red) that showed pronounced YAP amplification and distribution in both nuclei and cytoplasm in H314 carcinoma line in contrast to normal control OKF6 cells. Concurrently, suppression of p‐YAP and E‐cadherin was shown in the cancer cells. Quantitation of p‐YAP, YAP and E‐cadherin immunostaining in two cell lines is shown underneath (mean ± SEM, *P < 0.05, **P < 0.01 determined by the Student’s *t*‐test). (D) Confocal images for YAP, p‐YAP and desmoglein‐3 (DSG3) staining in the buccal carcinoma SqCC/Y1 cell line with a focus on the front migrating cells. Red arrowheads indicate the direction of cell migration. White arrows indicate enhanced nuclear YAP staining in the front migrating cells and white arrowheads indicate the co‐localisation of p‐YAP and DSG3 at the plasma membrane located opposite of the cell migrating front. Scale bars in C is 10 µm and D is 20 µm (including those in the inserts) (representative from two independent experiments).

To investigate the expression of YAP and its associated upstream regulators and downstream target genes in OSCC, we first evaluated the mRNA expression by quantitative PCR (qPCR) in a panel of ten authentic oral keratinocyte cell lines derived from two anatomic sites, that is BM and FOM. The clinical data of patients from whom cell lines were derived, as well as cell differentiation, are summarised in Table S1 [27, 29, 31, 33, 34]. Three normal (OKF4, OKF6 and SLC002), two dysplasia (D4 and D17‐TERT) and five carcinomas (H376, H314, H157, H413 and SqCC/Y1) were included in the study that had a broad coverage of clinical presentations (Table S1). Phase‐contrast microscopy revealed their morphological heterogeneity (Figs S1 and S2). In principle, four categories of morphologic features were identified in this study with characteristics as previous described [43], that is highly compact (HC), medium compact (MC), loosely associated/compact (LC) and fibroblast‐like and epithelial‐to‐mesenchymal (EMT) phenotype (F). These qualitative groups incorporated epithelial and mesenchymal aspects of the morphologies, including cellular shape and attachment to adjacent cells. For example, the ‘HC’ group contains cell lines that have a more typical epithelial morphology. In contrast, the ‘fibroblast‐like’ group contains mesenchymal‐like cell lines with more spindle‐shaped and make few contacts with adjacent cells. The other groups demonstrate a range of phenotypes between these extremes. Although both OKF4 and OKF6 are normal immortal cell lines, they showed a LC trait. D4, H376 and H157 were MC, SLC002 and two cancer lines, H413 and SqCC/Y1 displayed an HC trait, unexpectedly. D17‐TERT and H314 showed a typical EMT phenotype (F). Because YAP is sensitive to cell density [9], all cultures were extracted at approximately 60–70% confluent densities for analyses. Screening of 46 genes associated with the upstream and downstream activities of the Hippo pathway detected a general trend of YAP amplification in four OSCC lines except for H376 (Fig. 1A). In principle, three categories of genes were examined, that is the Hippo pathway (21 genes), cancer markers (8 genes) and intercellular anchoring junction‐associated (17 genes). For the Hippo pathway, apart from *YAP1* that showed upregulation in carcinoma cell lines (H314, H157, H413 and SqCC/Y1), *WWTR1*/TAZ was also overexpressed in H314, H413 and SqCC/Y1 plus D17‐TERT dysplasia line. Consistently, *TEAD 1‐4* were increased in carcinoma cells, with *TEAD1* being highly expressed in SqCC/Y1, *TEAD1‐4* in H314 and H413, and *TEAD2‐4* in H376. Other transcription factors (*TP73*, *ERBB4*, *TBX5 and RUNX3)* detected variations across cell lines, with *TP73* being notably upregulated in four cancer lines except for SqCC/Y1. However, except for SqCC/Y1, the two YAP target genes *CYR61*/*CTGF* did not show concurrent elevation with *YAP1* in carcinoma lines, especially in H157 that exhibited the highest level of *YAP1* (Fig. 1A). Intriguingly, a trend of upregulation in the majority of nine Hippo‐associated genes was found in four carcinoma lines except for H157 which retained a relatively low YAP activity (described below).

The second group of the genes was the cancer biomarkers, including three EMT transcription factors and five cell cycle progression regulators, all of which showed upregulation in carcinoma lines, indicating heightened cell proliferation as observed in cell cultures (Fig. 1A). Two lines with distinct morphologies, that is H314 with F phenotype and H413 with an HC trait, showed similar heightened expression of cancer biomarkers. The last group included both adherens junctions (AJ) and desmosomal (DSM) components. The Hippo pathway is partly regulated by E‐cadherin‐mediated homophilic adhesion that mediates epithelial cell differentiation and contact inhibition in line with Hippo signalling. Some components in the group, such as *CTNNA1* (encodes α‐Catenin) and *DSG3* are known as the components of the Hippo network [19, 44]. These genes are usually downregulated in the process of EMT and tumour invasion and metastasis. In general, there appeared a tendency of reduction in the cell junctional genes in dysplasia and cancer cells, but huge variations were observed in this group in OSCC lines (Fig. 1A). For instance, *CDH1* (encodes E‐cadherin) showed similar levels in all three normal cell lines as well as in H157 and SqCC/Y1 carcinoma lines. No reduction of *CDH1* was shown in H314 with characteristic EMT morphology. Other cell lines showed a decrease in *CDH1*. *CDH2*, a marker of EMT [45], did not show an increase in H314 either but was upregulated in D4, H376 and H157 with the highest detection in D17‐TERT. Two carcinoma lines H157 and SqCC/Y1 with MC presented less reduction of this group’s genes, especially in H157 that retained high levels of DSM components. In contrast, H413 with HC and a strong cancer signature showed a reduction of cell junctional genes except for *CTNNA1* and *NF2*. As mentioned above, H314 with an EMT trait exhibited high *CDH1*, *CTNNA1*, *NF2* and *PKP2/3*, all of which are the negative regulators of YAP [19, 44, 46, 47]. Therefore, there is no clear correlation between the cell morphology and YAP‐associated gene signature, including adhesion receptors. In addition, the heterogeneity of OSCC seemed to be independent of the tumour anatomic site.

Protein analysis showed some discrepancies from the qPCR results, for example little detection of TAZ was observed in H314 by western blotting (Fig. 1A,B). Three DSM proteins (Dsg2/3, Dp) and two AJ proteins (E‐cadherin, α‐Catenin) also showed some variations, as well as between cell lines derived from different tissue sites. While a gradual loss of DSG3 and E‐cadherin was detected in FOM cell lines during cancer transformation, this pattern was not seen in BM cell lines in which moderate E‐cadherin levels retained in three cancer lines in contrast to D17‐TERT that had little or no detection. Immunofluorescence confirmed the high YAP levels in H314 EMT‐like cells, with little phosphorylated YAP (p‐YAP) and E‐cadherin staining in contrast to OKF6 normal cell line with strong E‐cadherin signal coupled with attenuate YAP/p‐YAP (Fig. 1C), indicating a reciprocal negative regulation relationship [44]. Quantitation of YAP and E‐cadherin staining indicated clear reciprocal exclusive alterations of these two proteins (Fig. 1C). Changes in protein distribution and cell junctional structures for adhesion proteins in cancer cells were a consistent phenomenon. Finally, YAP staining in SqCC/Y1 cells indicated marked YAP nuclear localisation in the migrating front cells (arrows Fig. 1D) with concomitant p‐YAP restricted at the rear membrane regions in these migrating cells as well as in cells located inside of the colonies (arrowheads Fig. 1D). Additionally, expression of YAP/TAZ and p‐YAP along with DSG3 in different cell densities was analysed in OKF6 and SqCC/Y1 cell lines and showed density‐dependent increase of nuclear exclusion of YAP/TAZ in OKF6 and marked enhancement in the cytoplasmic expression of p‐YAP and membrane distribution of DSG3 in SqCC/Y1, respectively (Fig. S3). Furthermore, cell differentiation marker Involucrin was analysed by immunostaining coupled with E‐cadherin as well as western blotting. The results showed reduced or loss of Involucrin in the dysplasia and OSCC lines as opposed to normal control cells (Fig. S4), the finding agreed with a previous report [48] and suggesting that neoplastic transformation causes attenuation of the differentiation markers.

Overall, despite some variations, there was a general trend of elevated *YAP1/WWTR1* expression in OSCC cell lines which is consistent with other reports [3, 4]. Notably, the genes that encode the core components of the Hippo pathway also showed an increase in OSCC cell lines except for H157. These findings reflect the prominent feature of heterogeneity and dynamics in cancer evolution and transformation.

### 3.2 YAP is required for efficient oral cancer cell migration

**Fig. 2:**
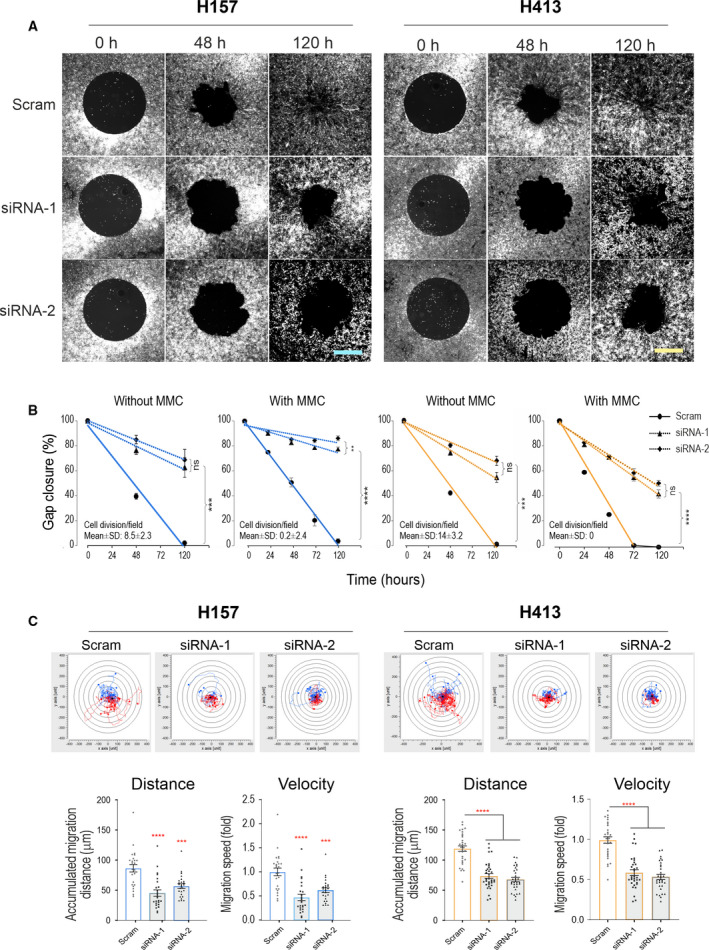
YAP knockdown results in marked suppression of collective cell migration in carcinoma cell lines. (A) Fluorescent images of cell migration towards the central circular cell‐devoid areas over time in H157 and H413 carcinoma cell lines. Cells were transfected with scrambled (Scram) control and YAP‐specific siRNA overnight before being plated in a 96‐well plate (Oris™ system). The images shown were cells treated without mitomycin c (MMC). The scores of cell mitotic events per 20× objective field in each condition are shown in the graphs. Scale bars, 100 µm. (B) Quantitation of cell migration overtime for up to 5 days. Cells were treated in the absence and presence of MMC, respectively (each plot was pooled from two independent experiments with four technical replicates (*n* = 12, pooled from three independent experiments, mean ± SEM). (C) Random cell migration tracking analysis in both cell lines treated with Scram control or YAP siRNA. The ability of cell movement was monitored 2 days later after siRNA transfection and MMC treatment by a time‐lapse microscope for 24 h. The images were acquired at the interval of 10 min in ten fields per 6‐well (*n* = 20, a representative from two independent experiments, mean ± SEM, Videos [Link], [Link], [Link], [Link]) (***P < 0.001, ****P < 0.0001 determined by one‐way ANOVA for B and C).

In light of emerging evidence that YAP is essential in cancer cell invasion and metastasis including HNSCC [3, 5, 8], we evaluated the role of YAP in the metastatic potential of OSCC lines by using transient RNA interference (RNAi, with two hits) to knockdown YAP in buccal carcinoma lines H157 and H413 that had high and moderate levels of YAP expression, respectively (Fig. 1A,B). After a pilot study to optimise the Oris™ Migration Assay conditions (Fig. S5), the ability of cell migration in various conditions was monitored in the absence and presence of mitomycin C (MMC) that abolished the effect of cell proliferation confirmed by DAPI staining (Fig. 2). Significantly, YAP depletion suppressed the capacity of cell migration in both H157 and H413 lines and this effect was observed consistently regardless of MMC treatment (*P* < 0.0001, Fig. 2A,B). Additionally, we performed the time‐lapse microscopy for random cell migration in both cell lines treated with scrambled or YAP siRNA that also showed remarkable repression of cell migration in all the YAP knockdown cells (*P* < 0.0001 Fig. 2C, Videos [Link], [Link], [Link], [Link]). Together, these findings suggest strongly that YAP is required for efficient OSCC cell migration.

### 3.3 YAP regulates cell proliferation and intercellular junction‐associated genes

**Fig. 3:**
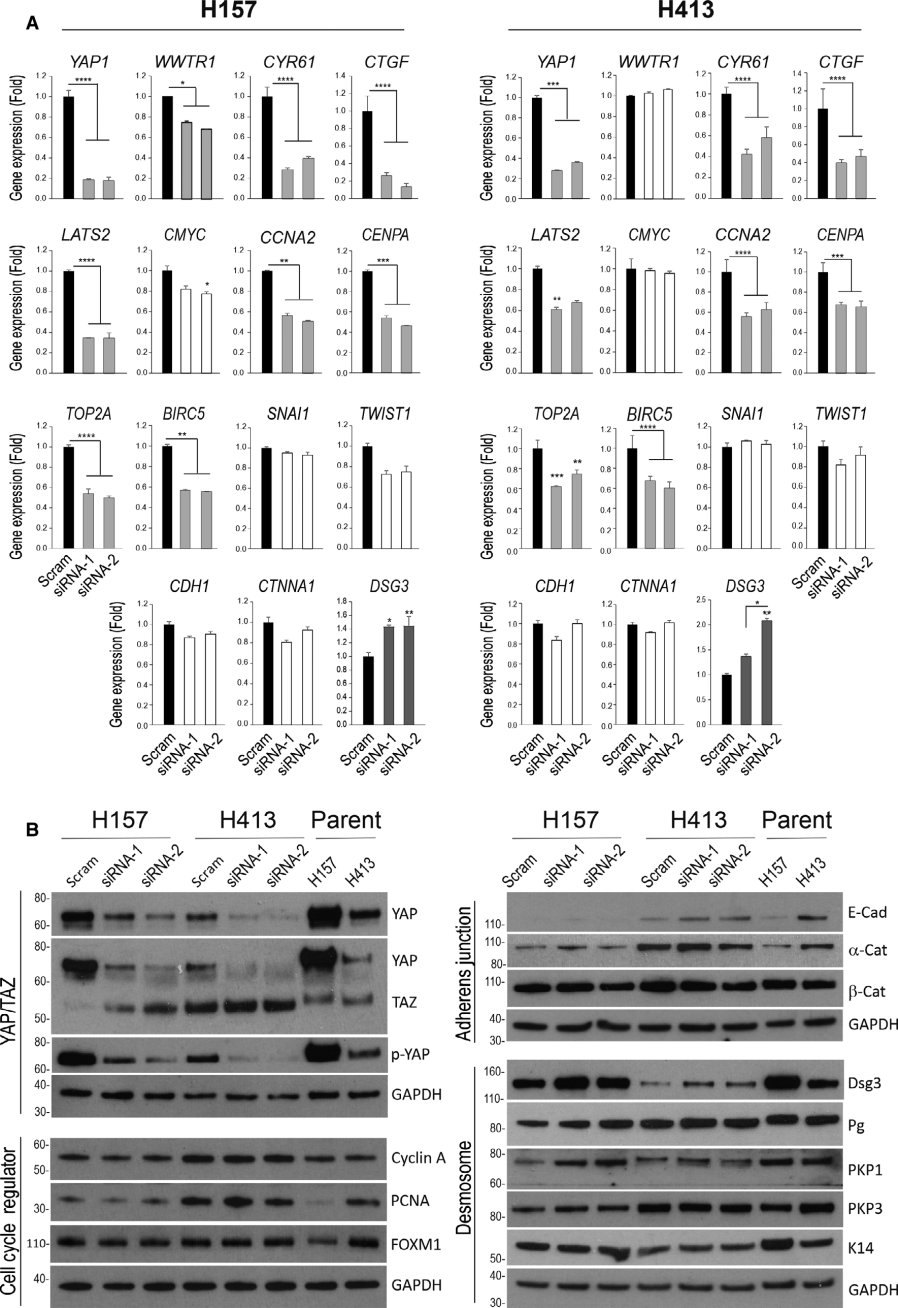
Effect of YAP knockdown on various genes in carcinoma cell lines. (A) qPCR data showed that YAP depletion resulted in a reduction of *WWTR1 (in H157 only*), two YAP target genes, five cell cycle progression regulator and survival genes but a marked increase in *DSG3* with a lesser effect on *CDH1* and *CTNNA1* that encode the AJ proteins E‐cadherin and α‐Catenin, in both cell lines (*n* = 12, pooled from three independent experiments with technical quadruplicate, error bar: SEM, *P < 0.05, **P < 0.01, ***P < 0.001, ****P < 0.0001 determined by one‐way ANOVA). (B) Western blotting analysis for the indicated proteins in both lines treated with scrambled control and YAP‐specific siRNAs alongside both parental cell lines (representative of two independent biological samples). GAPDH was used as a loading control. Note that YAP depletion caused a compensation of TAZ in H157 but not in the H413 line and showed increased expression in DSG3 gene and protein as well as other DSM proteins with little effect on E‐cadherin and α‐Catenin.

To investigate the molecular mechanism, we analysed a set of fifteen genes by qPCR, including two YAP targets, *WWTR1*, *LATS2*, four cell cycle regulators, survival gene *BIRC5*, two EMT (*TWIST1/SNAI1*) and three cell junctional components in both cell lines (Fig. 3A). Consistently, YAP depletion caused a marked reduction in *CYR61*/*CTGF* as well as *WWTR1* and *LATS2*. Except for *CMYC* that showed only a marginal reduction by YAP depletion, other cell cycle and survival genes were significantly downregulated by more than 30% in both lines compared to the respective controls. Unexpectedly, YAP knockdown had little effect on *SNAI1*/*TWIST1* that are involved in cell migration and metastasis [3]. In addition, almost no change was detectable in *CDH1* and *CTNNA1* except for *DSG3* that showed approximately 2‐fold induction by YAP depletion in both cell lines, the finding agreed with our recent report based on skin keratinocytes [26]. Western blotting confirmed sufficient knockdown of YAP, as well as p‐YAP, in both lines with concomitantly elevated expression of DSG3, Pg and PKP1/3, especially in H157 with DSG3 abundance (Fig. 3B), the finding also supported by immunofluorescence, along with E‐cadherin and α‐Catenin induced by YAP depletion (Fig. S6). Nevertheless, different results were observed for TAZ that showed moderate compensation in H157 but no change in H413 cells following YAP knockdown compared to the respective controls, suggesting compensation by TAZ only occurred in H157. Immunostaining for TAZ showed enhanced cytoplasmic translocation of TAZ, along with LATS1/2, in YAP depleted H157 cells in contrast to control cells that exhibited prominent TAZ nuclear staining (Fig. S6, data not shown). Notably, different levels of YAP and p‐YAP were presented in both parental cell lines with a 3‐fold increase of YAP/p‐YAP in H157, implying that the majority of YAP was phosphorylated and inactive protein (Fig. 3B). YAP knockdown also resulted in a subtle reduction of cell cycle regulators, especially in H157 line, but little influence in AJ‐associated E‐cadherin and α/β‐Catenin that was consistent with the qPCR data. No evident changes were observed in keratin K14 in both lines, and PKP1/Pg in H413 (Fig. 3B). Collectively, these results indicate that YAP deficiency affects the transcription program of cell proliferation, as reported in the literature [13, 49], with concurrent induction of DSM components such as DSG3 in OSCC cells.

### 3.4 Phenotypic variations of YAP dependency in OSCC cell lines

**Fig. 4:**
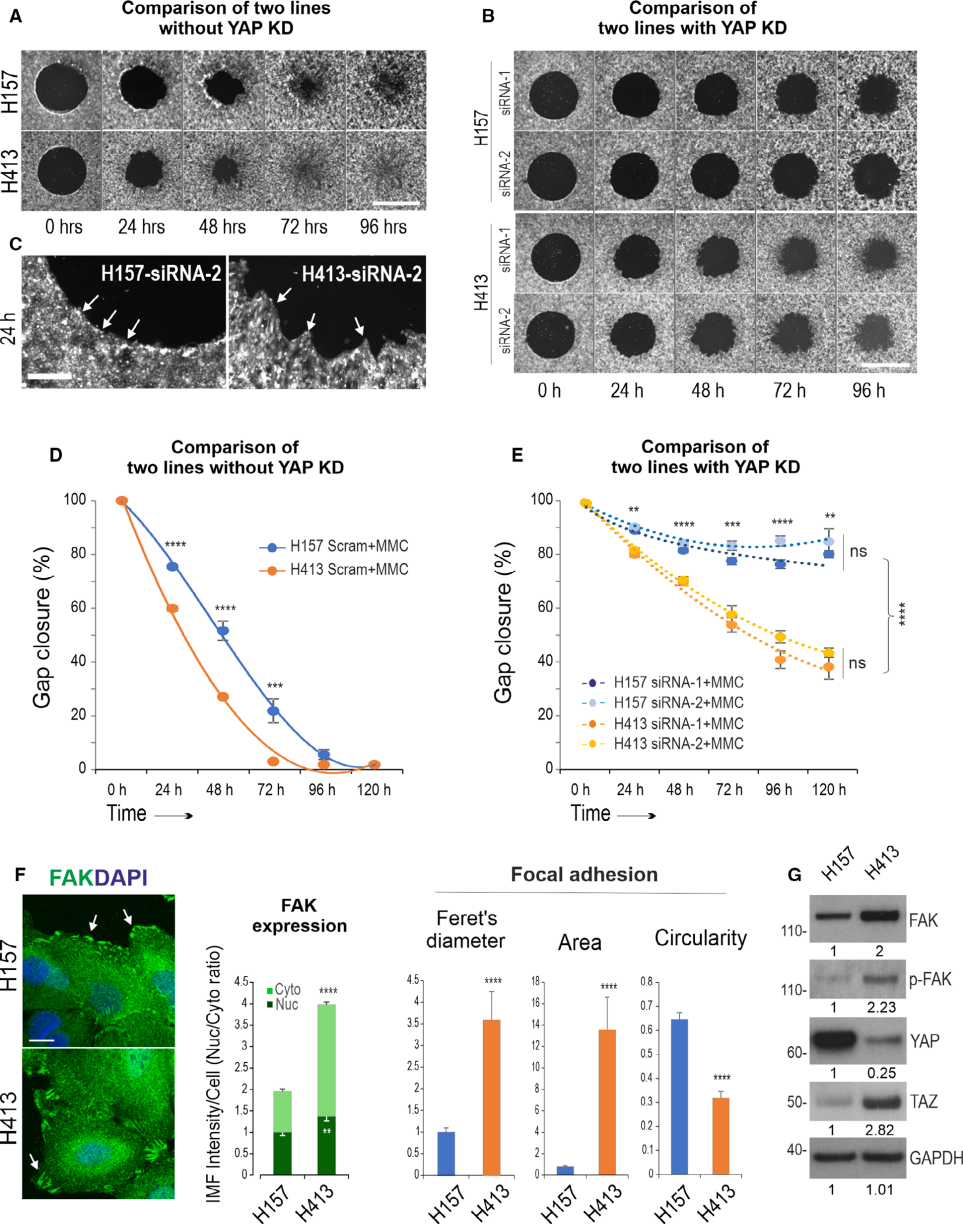
Distinct migratory phenotypes of YAP dependency in carcinoma cell lines. (A, B) Direct comparisons of cell migration in H157 and H413 cell lines treated with scrambled control siRNA and YAP RNAi, respectively. Cells were subjected to mitomycin c (MMC) treatment. The fluorescent images showed that the gap closures were faster in H413 than in H157 (the representative from three independent experiments). (C) Enlarged images focused on the migration front of YAP knockdown cells in two lines shown in A&B, indicating a linear curved line in H157 but marked irregularity in H413 cells (arrows). (D, E) Direct comparisons of the quantitation data in both lines without and with YAP knockdown, respectively (*n* = 8, pooled from two independent experiments with technical quadruplicate, error bar: SEM, **P < 0.01, ***P < 0.001, ****P < 0.0001 determined by the Student’s *t*‐test). (F) Morphological analysis of focal adhesion kinase (FAK)‐associated focal adhesions (FA) in both carcinoma lines including various parameters (*n* = 12, mean ± SEM, Student’s *t‐*test, **P < 0.01, ****P < 0.0001). Arrows indicate different FA streaks in two lines. (G) Western blotting for the indicated proteins (the representative from at least two independent experiments). GAPDH was used as a loading control. The densitometry values in the fold change are displayed underneath each blot. Scale bars in A and B is 1000 µm, C is 200 µm and F is 10 µm.

A recent study has identified the complexity of the Hippo‐YAP pathway in OSCC and highlights that only a subset of cancer lines in which the loss of YAP can be compensated for by its paralog TAZ [5]. Under this finding, close examination of our cell migration data found somewhat different migratory features in the two carcinoma lines. While the control H413 appeared to migrate faster than the control of H157 cells, such a difference became more revealing in both lines with YAP knockdown (Fig. 4A,B,D,E). YAP depletion in H157 showed almost complete abrogation of cell migratory in contrast to H413 that displayed only partial inhibition, suggesting a mixed phenotype of YAP dependence and independence in this line. In support, close inspection of enlarged images focusing on the cell migrating front depicted a linear circular line in H157 but a remarkable irregularity in H413 cells treated with YAP siRNA (arrows Fig. 4C). Quantitation demonstrated different efficiencies of cell migration in both lines treated with mitomycin C that essentially ruled out the effect of varied cell proliferation (Fig. 4D,E). This was further validated by Ki67 staining that showed no statistically significant difference between the two lines as Ki67 is an indicator of the number of cycling cells (data not shown).

Cells communicate with their environment through integrin‐mediated focal adhesions. Focal adhesion kinase (FAK), a nonreceptor tyrosine kinase, plays a vital role in regulating focal adhesion and migration signals, especially in tumour cells where its upregulation occurs in many cancer types, in particular in advanced‐stage solid tumours [50, 51]. Hence, we examined FAK and phospho‐FAK (Tyr397, p‐FAK) expression in the two cancer cell lines by immunofluorescence that found different morphologies of focal adhesions (FA), as well as FAK expression, with H413 displaying numerous FA streaks at the migrating front. In contrast, H157 showed a small rounded FA shape (arrows Fig. 4F). Quantitation for various parameters that reflect FA morphology revealed remarkable differences between the two lines (Fig. 4F). Given that FAK‐mediated signalling has been implicated in a variety of human cancers in tumour metastasis and progression, it was postulated that the enhanced cell migratory property in H413 could well be attributed to a YAP‐independent mechanism that might be associated with FA activity. It appeared that the two carcinoma lines in this study represented two distinct model systems, that is a YAP‐dependent H157 and a mixed phenotype of YAP‐dependent and YAP‐independent trait of H413 cells (described below).

### 3.5 YAP nuclear localisation, rather than its absolute expression levels, is associated with its activity in OSCC cell lines

**Fig. 5:**
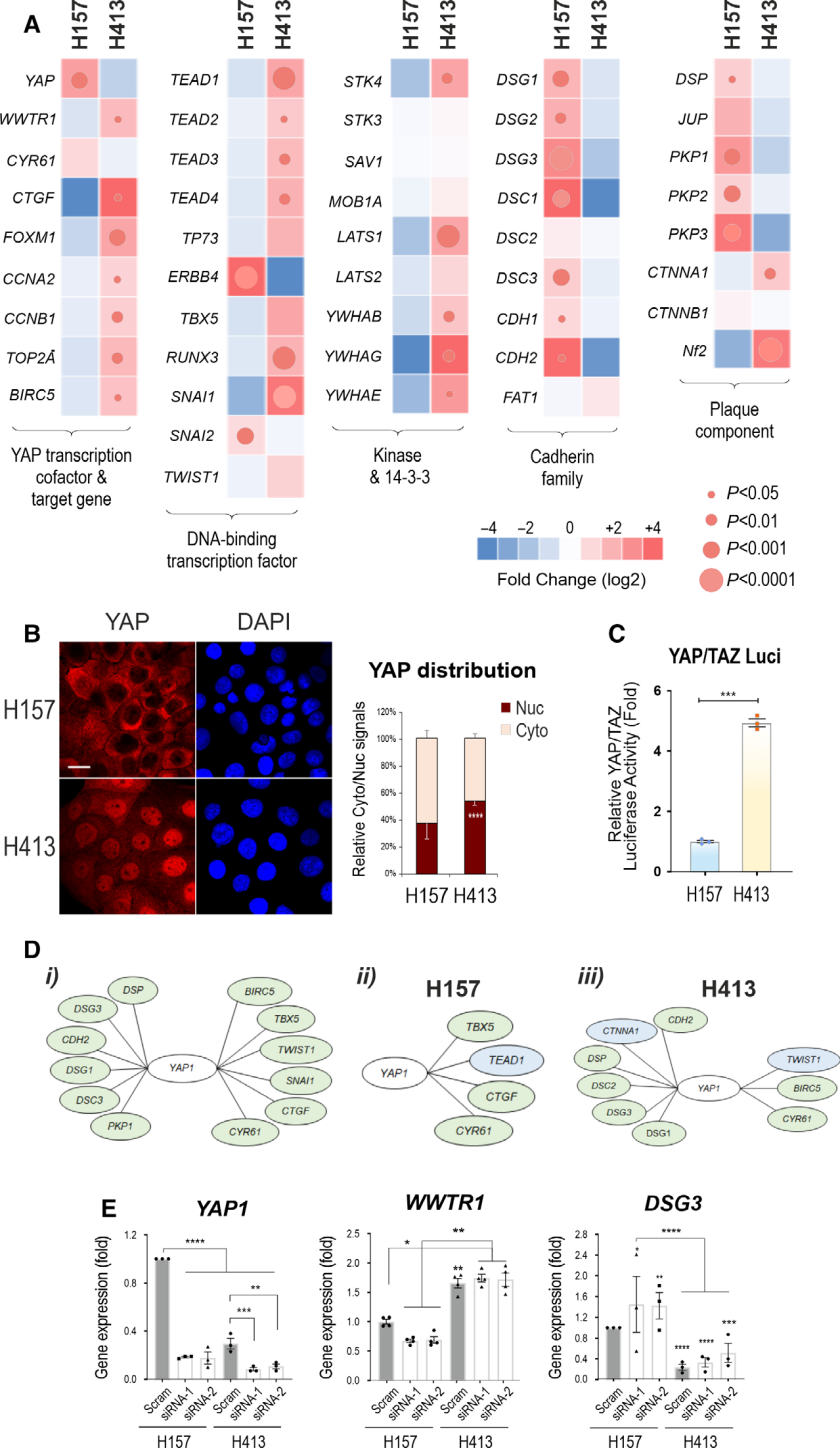
Distinct characteristics of OSCC lines in a correlation between YAP abundance and cell junctional components. (A) Heat map for direct comparison of the genes in five categories between H157 and H413 cell lines. Note that, despite the relatively low level of *YAP1*, H413 displayed a phenotype of active YAP signature with concomitant reduction of cell junctional genes except for *CTNNA1* and *NF2*. In contrast, a phenotype with an inverse trait was shown in H157 that lacked the YAP signature albeit *YAP1* expression was abundant. Besides, this line also presented elevated expression in many desmosome (DSM) genes. (B) Confocal microscopy of YAP staining in two carcinoma lines with the quantitation of relative nuclear (Nuc) versus cytoplasmic (Cyto) distribution shown on the right (*n* = 5, a representative of two independent experiments). (C) YAP/TAZ luciferase activity indicated a 5‐fold increase in H413 compared to H157 cells (pooled from three independent experiments). (D) Gene–gene interaction analysis identified the statistically significant genes which had a positive correlation with *YAP1*. The network map was generated based on the Pearson correlation coefficient. Genes shown in the green were *P* < 0.05 significantly correlated with *YAP1* and in blue were *P* < 0.01 significantly correlated with *YAP1*. Genes on the left of *YAP1* are potentially in the upstream Hippo pathway and those on the right are the known *YAP1* target genes. (D‐i) is the overall correlation network map based on the analysis of 46 genes in ten cell lines. (D‐ii, iii) are the networks in each carcinoma cell line. Note that several cell junctional genes were shown to be associated with *YAP1* only in the H413 line but not in H157. (E) Comparison of the qPCR data (normalised against the H157 control cells). Note that *YAP1* knockdown caused increased expression in *DSG3* in both lines (*n* = 8, two independent biological samples with technical duplicates) (Mean ± SEM, *P < 0.05, **P < 0.01, ***P < 0.001, ****P < 0.0001 determined by Student’s *t‐*test for A–D and for E determined by two‐way ANOVA for multiple comparisons).

To scrutinise the different phenotypes of the two carcinoma lines, we performed a direct comparison of the heat map in these two lines and observed their strikingly distinct gene signatures (Fig. 5A). Although elevated *YAP1* expression was shown in H157, the majority of the YAP‐associated genes, except for *CYR61*, *ERBB4* and *SNAI2*, was downregulated in this line in contrast to H413 with a high YAP signature. On the other hand, the cell junctional gene signals were relatively high in H157, except for *CTNNA1* (validated by immunostaining, Fig. S6), *NF2* and *FAT1* (YAP‐negative regulators). This finding was supported by protein analysis for Dsg2/3 and Dp as well as phospho‐YAP‐S127 (inactive‐YAP, refer p‐YAP hereafter) that showed elevated expression in H157. Calculation of the p‐YAP/total YAP ratio indicated 67% vs 47% in H157 and H413 lines, respectively, suggesting lower YAP activity in H157. To verify whether YAP nuclear localisation is associated with its activity, we performed confocal microscopy for YAP staining and detected predominant YAP nuclear localisation in H413 but in striking contrast, cytoplasmic distribution in H157 as supported by image quantitation (Fig. 5B). This finding was also correlated with high inactive p‐YAP in this line. Concordantly, the YAP/TAZ luciferase assay indicated a 5‐fold increase of luciferase reporter activity in H413 than H157 suggesting more active YAP/TAZ in H413 and that the nuclear YAP is correlated with its activity, albeit the expression level of YAP protein in this line is not abundant (Fig. 5C). Collectively, these results argue that YAP nuclear localisation, rather than the gene and protein abundance is the key indicator of its transcription activity.

Gene–gene interaction has been implicated in the functional relationship between two genes even though the two genes may not be involved in the same biological process or their encoded gene products may not necessarily interact with each other. To explore these properties, as well as their association with susceptibility to OSCC, we registered all the studied genes into MalaCards [52]. Additionally, we reviewed the publications for each gene that showed association with either SCC, HNSCC or OSCC (Table S2). As a result, a disease–gene association map with *YAP1* was generated (Fig. 5Di). Interestingly, this approach identified five DSM genes being potentially associated with *YAP1* that could be functionally correlated with OSCC. Regardless of cell lines, this analysis identified four upstream kinases (*STK3*, *SAV1*, *MOB1A* and *LATS1*), seven cadherins (*DSC1‐3*, *DSG1‐3 and CDH1*) and five junctional cytoplasmic components (*CTNNA1*, *NF2*, *PKP1*, *JUP* and *DSP*) that were significantly correlated with *YAP1*. Apart from *CDH1*, *DSG3*, *CTNNA1* and *PKP1* that are known to be functionally associated with YAP [19, 44], the other seven DSM genes remain largely unknown. Using Pearson’s correlation analysis, we constructed the networks with the genes that positively correlated with *YAP1* in H157 and H413 (Fig. 5Dii,iii). The *YAP1* target genes and its DNA binding partners [9, 10] are shown on the right. In addition to TEAD family members, *TBX5* is known to be correlated with *YAP1* by forming a complex with YAP and β‐Catenin in initiating the expression of *BIRC5* that encodes Survivin and is crucial for cancer cell survival [11]. The EMT transcription factor *TWIST1* was also identified to have a correlation with *YAP1* [6]. The genes on the left of *YAP1* could potentially activate or inhibit the Hippo pathway, leading to the inhibition or activation of *YAP1*, respectively. Among six junctional genes (*CDH2*, *CTNNA1*, *DSP*, *DSC2*, *DSG1/*3), three DSM genes remain largely unknown to be associated with YAP function. Notably, distinct maps were generated for the two cancer lines. While no link between *YAP1* and junctional components was detected in H157, six junctional genes were associated with *YAP1* in H413. As illustrated in Fig. 5A,D, the DSM genes exhibited a negative correlation with *YAP1*, as exemplified in H413 cells.

In addition to FAK, TAZ is also known to function as a *bona fide* oncogene by promoting tumour cell proliferation, EMT and chemoresistance in OSCC and its enforced overexpression confers a phenotypic transition from noncancer stem cells to cancer stem cell‐like cells [53]. Direct comparison of the gene signatures revealed *WWTR1* abundance in H413 in contrast to H157, with no marked reduction induced by YAP knockdown (Fig. 5A,E). Furthermore, although a marginal increase of *DSG3* was detected in H413 cells with YAP knockdown its overall expression levels remained low, that is less than 50% of H157 control cells, the results that were validated by western blotting and immunofluorescent analyses (Fig. 3, Fig. S6). These findings led us to hypothesise that both the heightened TAZ and low DSG3 abundance in H413 might attribute to the residual cell migratory property in YAP knockdown cells.

### 3.6 Diverse contributions of YAP and TAZ to sufficient cell migration in OSCC cell lines

**Fig. 6:**
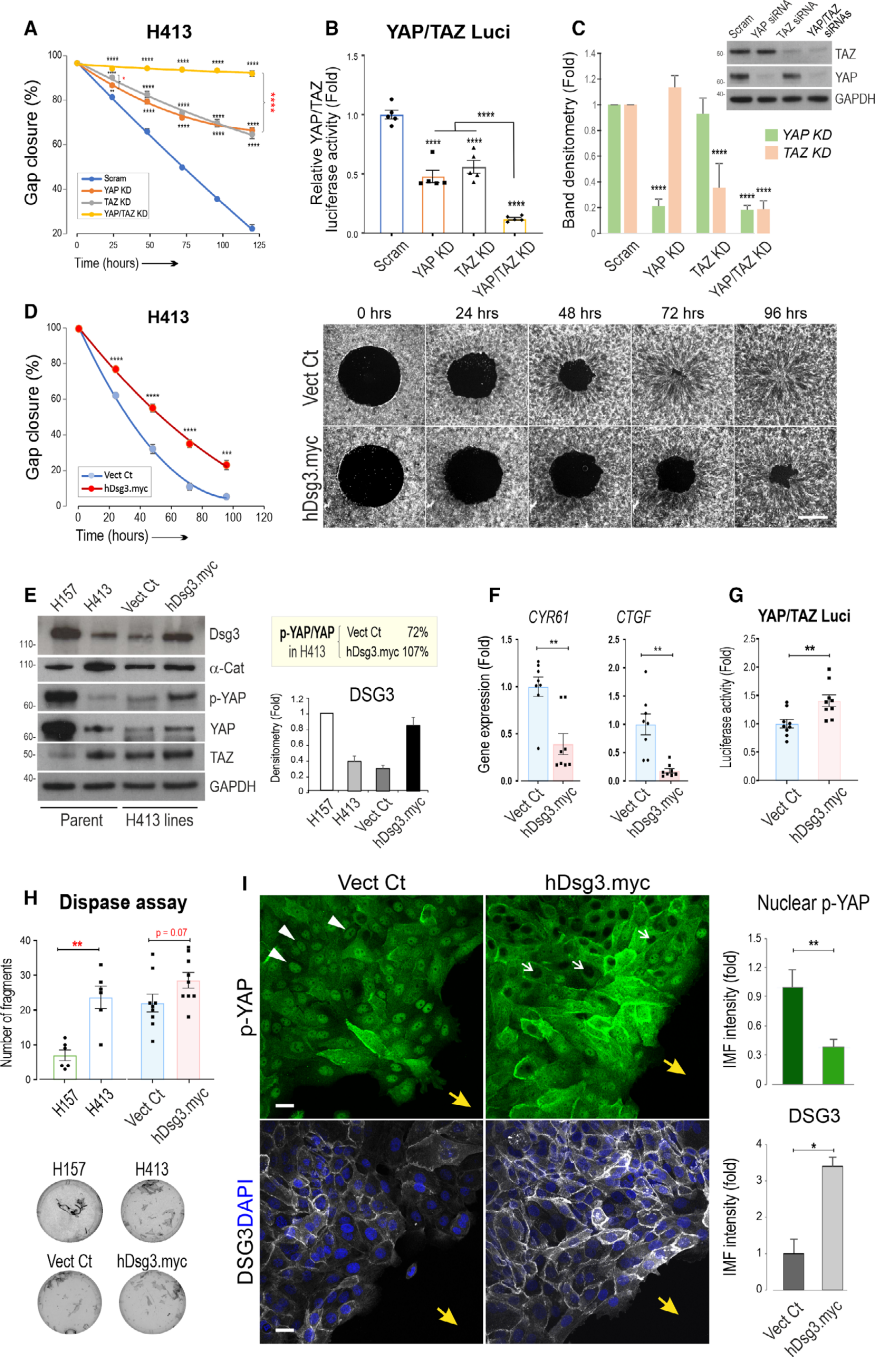
A mixed phenotype with both YAP and TAZ dependency in cell migration in the H413 carcinoma cell line in which overexpression of DSG3 restricts the capacity of collective cell migration. (A) Quantitation of Oris™ Migration Assay in H413 cells treated with scrambled control siRNA, or with single and double knockdown of YAP and TAZ, respectively. Cells were transfected with siRNAs overnight before being plated in Oris™ 96‐cell plate. Cells were treated with mitomycin c (MMC) for 3 h before the assay (data shown were representative of three independent experiments, with four technical replicates). (B) YAP/TAZ luciferase assay in H413 cells with single and double YAP and TAZ knockdown (*n* = 3 biological replicates). (C) Western blotting and densitometry analysis in H413 cells with single and double YAP and TAZ knockdown (*n* = 3 biological replicates). (D) Oris™ Migration Assay of H413‐Vect Ct and hDsg3.myc stable lines indicating inhibition of cell migration in hDsg3.myc cells, compared to Vect Ct line (*n* = 8, pooled data from two out of three independent experiments/batches of the cell lines, with technical quadruplicate). (E) Western blots with the indicated antibodies in parental H157 and H413, as well as in H413‐Vect Ct and hDsg3.myc stable lines. Note that ectopic DSG3 expression in hDsg3.myc cells restored its expression level close to that of H157 cells and caused a marked increase in p‐YAP and to a lesser degree in YAP, as well as TAZ and α‐Catenin. The DSG3 band densitometry was shown in the bar chart (*n* = 3 biological replicates). (F) The qPCR analysis of two *YAP1* target genes (*n* = 2 biological replicates, each with four technical replicates) and (G) YAP/TAZ luciferase assay in H413‐Vect Ct and hDsg3.myc lines (*n* = 3 biological replicates). (H) Dispase cell dissociation assay in four conditions, that is parental H157 and H413 cells and H413‐Vect Ct and hDsg3.myc stable lines. The images on the left were the fragments of epithelial sheets induced by mechanical forces in this assay, with the quantitation of fragments shown on the right (*n* = 9, pooled from three independent experiments with technical triplicate). Note that there appeared a trend of increase in fragmentation in hDsg3.myc cells compared to Vect Ct. On the other hand, H157 cells showed significantly less fragments than parent H413, and no difference was detected between parent H413 and H413‐Vect Ct lines. (I) Confocal images and quantification of p‐YAP nuclear and Dsg3 staining in the cell migrating front in Vect Ct and hDsg3.myc lines (the representative from 2 independent experiments, yellow arrows indicate the direction of cell migration, white arrowheads indicate p‐YAP nuclear staining and white arrows indicate cells with the absence of p‐YAP nuclear signals) (Mean ± SEM, *P < 0.05, **P < 0.01, ***P < 0.001, ****P < 0.0001 determined by Student’s *t‐*test except for A and B determined by two‐way ANOVA). Scale bars, 10 µm.

To investigate the aforementioned possibilities, first, we performed the cell migration assay in H413 cells with *WWTR1*/TAZ knockdown [19] alongside YAP single knockdown and YAP/TAZ double knockdown. Significantly, only double YAP/TAZ knockdown resulted in substantial inhibition of cell migration compared to YAP or TAZ knockdown alone (Fig. 6A) suggesting the residual migratory property of YAP knockdown cells of H413 was attributed to TAZ activity. Western blotting and YAP/TAZ luciferase analyses demonstrated significant depletion and inactivation caused by YAP and TAZ single or double knockdown (Fig. 6B,C). These results demonstrated both YAP and TAZ are required in controlling OSCC cell migration and exhibited partially overlapping activities as exemplified in the two model systems of H413 and H157 cell lines.

### 3.7 Dsg3 negatively regulates YAP by inducing phosphorylated YAP expression in OSCC cells

Next, we examined the role of DSG3 in OSCC cell migration. Our previous study based on vulvar carcinoma A431 cell line has implicated that DSG3 promotes cancer cell migration and invasion [22]. Using the same gain‐of‐function approach [20, 22], we generated a stable H413 line with transduction of hDsg3.myc alongside a matched control line with transduction of empty vector pBABE.puro (Vect Ct). After drug selection, hDsg3.myc and Vect Ct cell lines were subjected to Oris™ Cell Migration and scratch wounding assays as well as analyses by other techniques. Reproducibly, overexpression of DSG3 (three different batches being tested) resulted in a marked inhibition of cell migration compared to Vect Ct (Fig. 6D, Fig. S7A) suggesting DSG3 suppresses cell motility. This finding agreed with the observation in YAP knockdown cells that exhibited upregulation of DSG3 with inhibition of cell migration (Figs 2 and 3). Furthermore, time‐lapse microscopy was performed to monitor cell migration for 15 h in the scratch wounding assay (Videos S5 and S6) and also the sparse cultures, both of which were pretreated with MMC that ruled out the effect of cell proliferation. The cell tracking for the random cell migration showed a consistent finding, that is hDsg3.myc cells exhibited a marked reduction of cell migration compared to Vect Ct (Fig. S7B, Videos S7 and S8). In parallel, we also analysed DSG3 knockdown in H157 cells that had high DSG3 expression and monitored their ability of migration alongside the cells with double knockdown of YAP/DSG3. However, with two different DSG3 siRNA hits, little or no effect was observed on cell migration compared to control except for cells with combination of YAP depletion (Fig. S8A). Western blotting showed that DSG3 knockdown resulted in decrease of YAP and subtle changes in TAZ and α‐Catenin with marginal induction of E‐cadherin (Fig. S8B). Together, these data suggest that overexpression of DSG3 in OSCC cells blunted collective cell migration, with DSG3 depletion showing no obvious effect.

Western blotting analysis indicated that ectopic expression of DSG3 restored the protein level similar to that of H157 cells, leading to increased expression of p‐YAP, as well as total YAP to a lesser extent, and also α‐Catenin (Fig. 6E). Calculation of the p‐YAP/total YAP ratio in Vect Ct and hDsg3.myc cell lines indicated 1.5‐fold increase in DSG3 overexpressing cells (Fig. 6E). In accordance, DSG3 silencing resulted in a reduction of YAP/p‐YAP (Fig. S8B), the results agreed with our previous report [19]. Collectively, these data argued that DSG3 likely inhibits YAP activity by preferentially mediating the expression of inactive p‐YAP. This conclusion was corroborated by qPCR analysis that showed a marked reduction of *CTGF*/*CYR61* in hDsg3.myc cells (Fig. 6F). However, paradoxically, the YAP/TAZ luciferase assay identified a moderate but statistically significant increase of their transcription activity in hDsg3.myc cells compared to Vect Ct (Fig. 6G) suggesting likely that an additional ‘noncanonical’ YAP/TAZ function was triggered by DSG3 overexpression because a marginal increase of TAZ was also detected in DSG3 overexpressing cells (Fig. 6E).

Since elevated α‐Catenin was detected in hDsg3.myc line, we performed the Dispase cell dissociation assay [26, 42] in both cell lines, alongside two parental H157 and H413, to determine whether overexpression of DSG3 enhances cell–cell adhesion in OSCC cells. However, the results showed that DSG3 overexpression in H413 did not enhance cell–cell adhesion at all, but instead resulted in a trend for weakening cell cohesion (*P* = 0.07 Fig. 6H). In contrast, the H157 cell line that retained high levels of DSG3 and other junction assembling proteins showed statistically significantly less fragmentation compared to H413 cells, indicating stronger cell–cell adhesion strength. These results implied that DSG3 may not act as a *bona fide* cell adhesion protein *per se*, but instead exerts a function in regulating Hippo signalling by inducing p‐YAP expression that participates in the process of contact inhibition of locomotion in OSCC cells. Furthermore, immunostaining for p‐YAP in the collective migrating cells of the monolayer in Vect Ct and hDsg3.myc cell lines detected numerous nuclear signals in Vect Ct cells but in contrast, predominant membrane and cytoplasmic localisation with concurrently decrease of p‐YAP nuclear signals were observed in hDsg3.myc cells with DSG3 overexpression (Fig. 6I), indicating that enhanced expression of DSG3 promoted p‐YAP nuclear exclusion and cytoplasmic translocation via forming a protein complex with DSG3 [19].

**Fig. 7:**
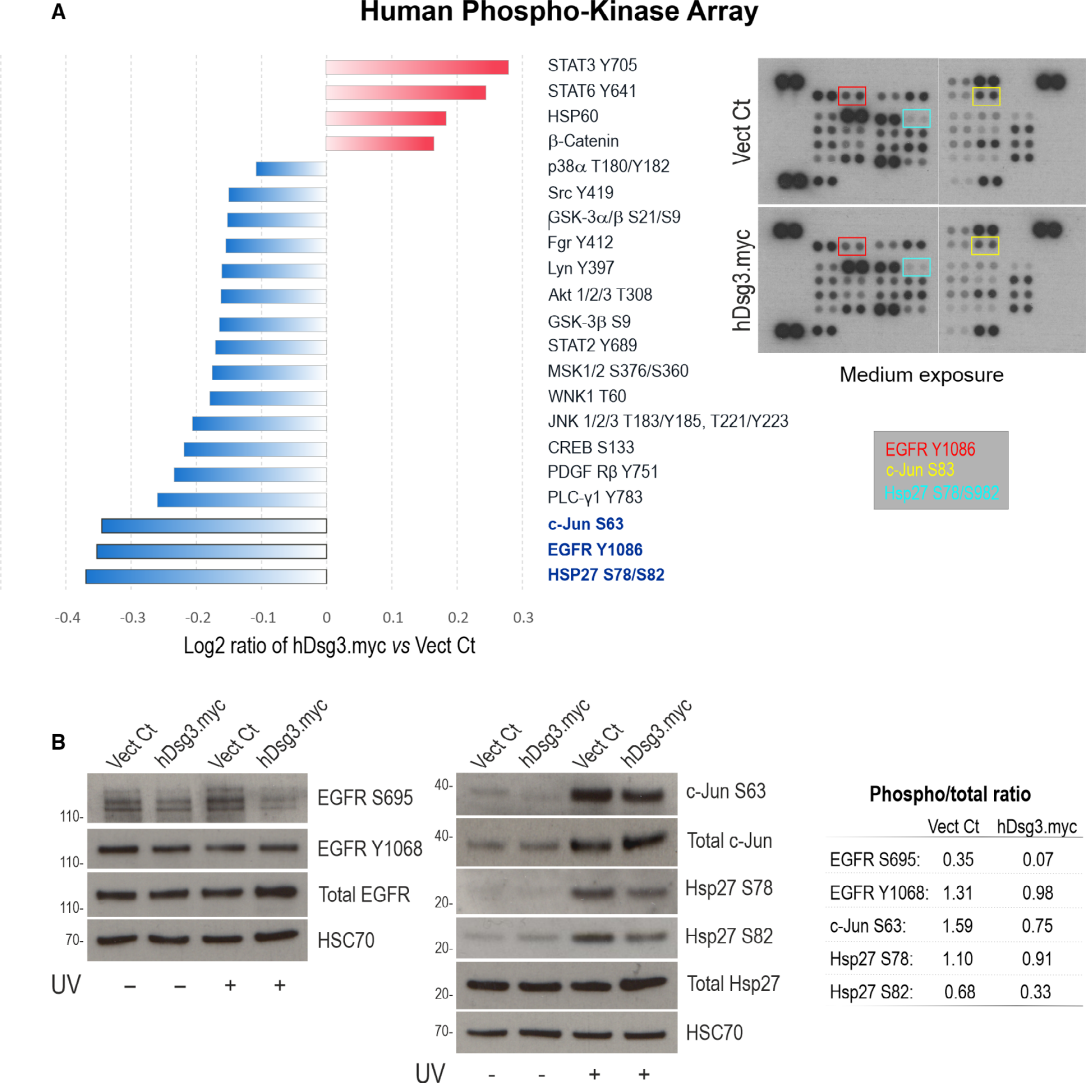
Human phospho‐kinase array identifies inhibition in EGFR, Hsp27 and c‐Jun by DSG3 overexpression. (A) Human phospho‐kinase array data presented as the log 2 ratio of fold change in H413‐hDsg3.myc against Vect Ct. The cut‐off values were between > −0.1 and < 0.1. A significant decrease in the phosphorylation of epidermal growth factor receptor (EGFR) Y1086, transcription factor AP‐1 (c‐Jun) S63 and heat shock protein beta‐1 (Hsp27) S78/S82 was observed among others shown (*n* = 1 biological sample and 2 technical duplicates). (B) Western blotting validation with the indicated antibodies and the ratio of phospho *vs*. total proteins are shown on the right (*n* = 2 biological replicates). Reduced expression of EGFR Y1086 in hDsg3.myc cells were also observed compared with Vect Ct (data not shown due to the high background).

To further address the mechanistic insight, we performed the Human Phospho‐kinase Array study in H413‐Vect Ct and hDsg3.myc cell lines and cells were allowed to grow to approximately 80–90% confluence. As shown in Fig. 7, we identified a reduction of various phospho‐kinases in DSG3 overexpressing cells, in particular EGFR Y1086, Hsp27 S78/S82 and c‐Jun S63 that were validated by western blotting in cells treated with and without UVB radiation (30 mJ·cm^−2^). We also detected suppression of EGFR S695, but not Y1068, in DSG3 overexpressing cells compared to Vect Ct (Fig. 7B). Only STATS3 Y705, STATS6 Y641, HSP60 and β–Catenin showed an increase in hDsg3.myc cells in this array study. Collectively, these data suggest that DSG3 inhibits YAP and promotes p‐YAP expression indirectly via inhibition of EGFR signalling and its downstream Hsp27 and c‐Jun in oral keratinocytes, the findings agreed with what has been characterised for DSG3 from pemphigus research [54].

Finally, we evaluated the effect of DSG3 overexpression on FAK/p‐FAK in their expression and morphological changes of FA in H413‐hDsg3.myc and Vect Ct lines. Although no evident changes were observed by western blotting analysis (data not shown), confocal microscopy revealed suppression of FA in hDsg3.myc cells compared to the Vect Ct (Fig. S9). Furthermore, fluorescent staining for F‐actin showed hDsg3.myc cells presented less actin stress fibres than Vect Ct cells with an overall reduction of actin staining signals. On the other hand, knockdown of DSG3 resulted in enhanced F‐actin stress fibres (possibly with enhanced cytoskeletal tension) (Fig. S10). Collectively, these results suggest that DSG3 confers p‐YAP‐mediated inhibition of cell locomotion via suppressing the FA formation and signalling in OSCC cells.

## 4 Discussion

The Hippo signalling pathway has recently been shown to play a major role in shaping tumour progression and to have a strong correlation with patient survival time [55]. Despite the heterogeneity and limitation in the number of cell lines in this study, there appeared a general trend of YAP overexpression in the evolution of oral carcinogenesis, a finding that agrees with reports by others [3, 5, 6]. In addition to *YAP1*/*WWTR1*, the majority of Hippo components were also amplified in OSCC lines that is likely caused by a negative feedback loop upon YAP activation [56]. YAP/TAZ activation and concomitant enhancement of TEAD4 are implicated in the progression of HNSCC [6]. YAP/TAZ‐activated genes involved in cell cycle progression and survival (*BIRC5*) that are increased with OSCC tumour grade or stage were also identified here [57]. Increased cancer biomarkers in OSCC lines indicate the authentic nature of these resources. Although the variations were observed in cell junctional genes, there still seemed a general loss in dysplasia and cancer cells. No clear correlation between cancer evolution and the cell morphologies were found in keratinocyte culture. Variations in E‐cadherin between FOM OSCC (loss of E‐cadherin) and BM OSCC (retaining E‐cadherin expression) cell lines remain not fully understood. It was thought that this may not solely be attributed to cell differentiation but rather caused by other cellular functions of E‐cadherin whose expression has additionally been linked to cell activities such as invasiveness reduction, growth inhibition, apoptosis and the cell cycle arrest. Studies on various cancers have shown that these different cellular functions of E‐cadherin are interdependent [58]. Although the OSCC cell lines used in this study were described to be moderately or well‐differentiated (Table S1), the loss of E‐cadherin in two FOM cell lines might reflect the fast‐growing feature in H376 or EMT in H314 as observed in our tissue cultures. The same analogy could apply to DSG3 as it has been identified as a pro‐survival protein and biomarker in squamous cell carcinoma [21, 59].

YAP is known as a key player in cell migration via suppression of E‐cadherin‐mediated AJs and enhancement of EMT [39, 60, 61, 62]. YAP is also considered as a biomarker for metastasis and resistance to EGFR inhibitors (*Gefitinib*, *Cetuximab*) in HNSCC [3, 4]. Cell migration is an essential process for normal tissue function as well as cancer progression and metastasis which accounts for the vast majority (90%) of cancer‐related deaths. Although the Hippo downstream effectors YAP/TAZ are well characterised, the upstream regulators remain incompletely elucidated. This study has identified six DSM genes (*DSG1/*3, *DSC2/3*, *PKP1*, *DSP*), in addition to *CDH2* and *CTNNA1*, which are potentially associated with *YAP1* and exhibited a mutually exclusive dependency. Apart from *DSG3* and *PKP1*, the other four DSM genes remain unknown to be linked with *YAP1* function. Thus, the study unravels several DSM components that may potentially play a role in the Hippo pathway to control YAP/TAZ function, similar to α‐Catenin and E‐cadherin‐mediated AJs, especially in oral keratinocytes with abundant DSG3 expression.

YAP expression levels may not be correlated to its activity in cancer cells. YAP nuclear translocation accompanied by decreased levels of p‐YAP is indicative of YAP function and resistance in OSCC cell lines, rather than YAP abundance [6, 63]. Our study has provided direct evidence with two model systems of OSCC cells and demonstrated that H157 had abundant YAP/p‐YAP but low YAP activity. In contrast, H413 cells exhibited an opposite trait with low levels but high YAP nuclear activity. Concordantly, predominant YAP cytoplasmic localisation was detected in H157 but its nuclear localisation in H413. Furthermore, a reciprocal exclusive relationship in six junctional genes with *YAP1* was detected only in H413 line but no link was identified in H157. Elevated expression of several Hippo components and the genes associated with EMT was detected exclusively in H413. For cell migration, we identified variations in YAP dependence with partially overlapping activity of YAP between two lines. While H157 exhibited a YAP‐dependent phenotype, H413 manifested a mixed phenotype of YAP‐dependent and independent features, with the latter being attributed to TAZ activity. Therefore, YAP depletion in H157 cells substantially blunted cell migration ability, but this was not the case in H413 unless both YAP and TAZ were knocked down. Importantly, our findings provide a unique mechanistic paradigm for the control of collective cell migration in OSCC cells and argue that DSG3 acts as an important regulator to modulate p‐YAP expression and inhibit canonical YAP nuclear activity. Thus, analogous to α‐Catenin, DSG3 functions as an important signalling molecule in the Hippo signalling network to govern cellular contact inhibition of locomotion beyond its role in cell adhesion. YAP depletion resulted in upregulation of DSG3, also observed in another report [26]. This mechanism seemed to be involved in suppression of FAK‐mediated FA that has been shown to control YAP mechanotransduction [64]. We also found DSG3 acts in response to mechanical forces in keratinocytes [19]. Thus, there could be another level of mutual regulation between cell–matrix and cell–cell interaction in mechanosensing. Specifically, DSG3 also exhibits a function different from α‐Catenin (discussed below) since it has been identified as a potential oncogene in promoting cancer progression and metastasis [20, 21, 22, 59, 65, 66]. Overall, our study reveals the complexity and variations of the Hippo‐YAP pathway in OSCC lines which agrees with another report [5].

**Fig. 8:**
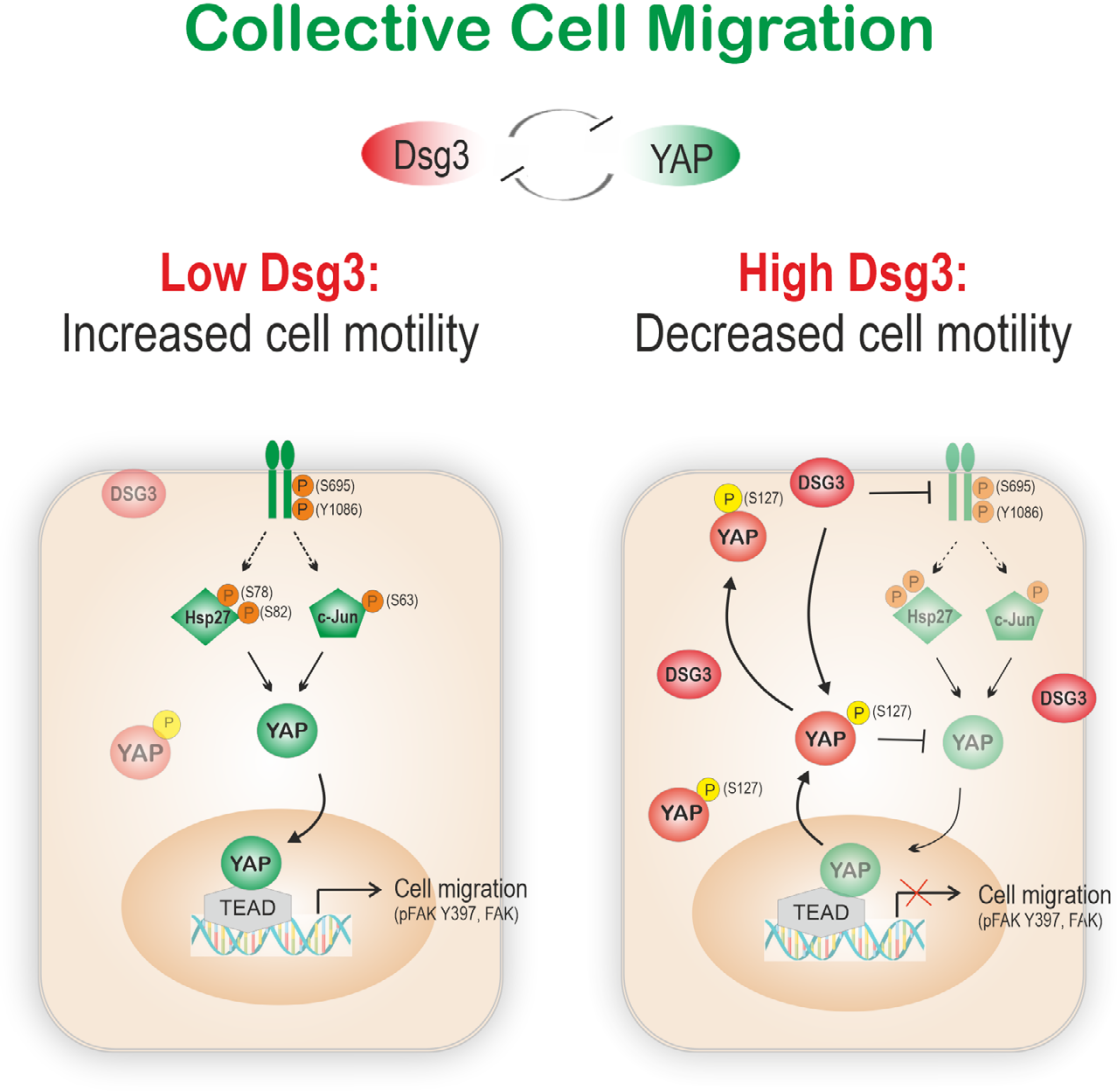
Proposed model on how elevated DSG3 expression contributes to the suppression of YAP and its mediated collective cell migration. In OSCC cells, mutual exclusive regulation occurs between DSG3 and YAP. In cells with low DSG3, YAP is activated via EGFR‐mediated signalling that involves phosphorylation of EGFR S695/Y1086 and downstream Hsp27 S78/S82 and c‐Jun S63 and thereby, YAP nuclear localisation and gene transcription associated with cell migration involved FAK and p‐FAK Y397. In contrast, in cells with high DSG3, EGFR signalling is inhibited by DSG3 that elicits YAP inhibition and phosphorylation. As a result, p‐YAP S127 is recruited from the nucleus to the cytoplasm as well as towards the plasma membrane, leading to contact inhibition of cell locomotion and attenuated collective cell migration.

Our studies [19, 20, 22, 23, 24, 26] suggest that DSG3 acts as a pleiotropic protein depending on the cell context as well as the experimental setting or migration modes. We demonstrate that, in OSCC cells, DSG3 abundance can restrict cell migration ability in collective cell migration, as well as in random cell migration. In this scenario, DSG3 negatively regulates YAP nuclear activity by enhancing expression and cytoplasmic translocation of p‐YAP and even at the membrane regions [19], leading to contact inhibition of locomotion. Mechanistically, we showed that DSG3 exerts a function by inhibiting the EGFR signalling pathway that involves both Hsp27 and c‐Jun to activate YAP downstream. Specific EGFR tyrosine residues play a key role in determining epithelial cell chemotaxis and restitution [67], an event that requires Src family kinase‐dependent p38MAPK activation [68]. We detected that EGFR Y1086/S695 but not Y1068 were modulated by DSG3. Hsp27 is a target of p38MAPK [54]. Consistently, increased expression of Hsp27 is shown to be correlated with the YAP gene signatures and concurrent reduction of p‐YAP in the lung and prostate tumours, as well as in the invasive breast cancer [69]. Furthermore, AP‐1 (dimer of Jun and Fos proteins) is reported to synergistically activate YAP gene transcription by forming a complex with YAP/TAZ/TEAD in the nucleus [70]. Thus, inhibition of this EGFR signalling cascade by DSG3 can have an impact on YAP activity and phosphorylation. Therefore, DSG3 may participate in Hippo signalling by fine‐tuning YAP activity depending on cell–cell compact or polarisation. This theory can also explain what we have reported previously that DSG3 overexpression in A431 carcinoma cells promotes cell migration and invasion since in this model system, DSG3 activates Src signalling, among others, and downregulate E‐cadherin‐mediated adherens junctions [20, 22, 24]. Hence, our studies provide a unique working model for DSG3 in regulating the Hippo‐YAP pathway and submit mutually exclusive regulation between DSG3 and YAP in oral cancer cells (Fig. 8). Other mechanism by which DSG3 directly induce p‐YAP expression and the membrane sequestration is not ruled out either [19]. Paradoxically, we also observed that DSG3 exhibited a function in promoting YAP/TAZ luciferase activity that differs from α‐Catenin in regulating YAP [49]. In accordance, DSG3 silencing resulted in YAP reduction [19]. We reason that this could be due to a noncanonical YAP nuclear function or the action of TAZ which warrants further investigation. A previous study failed to detect hyperplastic changes in mice with Mst1/2 depletion nor in human HaCaT keratinocytes with LATS1/2 knockdown [49]. Since DSG3 is identified as a versatile signalling molecule and can influence various signalling pathways [15, 19, 20, 21, 22, 23, 24, 38, 59, 71], it may function as a potential key component in Hippo signalling. Further study is needed in deciphering its role in the Hippo pathway.

## 5 Conclusion

In conclusion, this *in vitro* study showed a trend of YAP overexpression in OSCC cells. However, YAP abundance may not be indicative of its activity. Thus, caution needs to be taken to draw a conclusion based on YAP abundance. Two distinct OSCC cell model systems in YAP expression and activity were identified here highlighting that YAP activity is predominantly associated with its nuclear localisation. Furthermore, the study reveals mutually exclusive dependence between YAP activity and the desmosomal components and sheds light that DSG3 acts as an important component of the Hippo pathway in the control of collective OSCC cell migration [6].

## Conflict of interest

The authors declare no conflict of interest.

## Author contributions

USA performed conceptualisation, data curation, formal analysis, validation, investigation, visualisation, methodology, writing—original draft, and writing—review and editing; EKP performed resources, supervision, conceptualisation, and writing—review and editing; HW involved in conceptualisation, data curation, formal analysis, resources, validation, investigation, visualisation, methodology, supervision, project administration, writing—original draft, and writing—review and editing.

### Peer Review

The peer review history for this article is available at https://publons.com/publon/10.1002/1878‐0261.13177.

## Supporting information


**Fig. S1.** Phase‐contrast images of five oral floor of mouth keratinocyte cell lines.

**Fig. S2.** Phase‐contrast images of five oral buccal keratinocyte cell lines.

**Fig. S3.** YAP exhibits cell density‐dependent subcellular translocation from the nucleus to the cytoplasm with concomitant elevated p‐YAP expression.

**Fig. S4.** The differentiation marker Involucrin staining shows reduced or loss in oral dysplasia and carcinoma cell lines.

**Fig. S5.** A time‐course study of Oris™ migration assay in H157 cell line.

**Fig. S6.** YAP knockdown causes increased cytoplasmic localisation of TAZ in the H157 line and enhanced expression of DSG3, E‐cadherin and α‐Catenin in both lines.

**Fig. S7.** Overexpression of DSG3 inhibits both the collective and random cell migration in the OSCC H413 cell line.

**Fig. S8.** DSG3 knockdown causes a reduction of YAP and p‐YAP but with no evident effect on collective cell migration in OSCC cells.

**Fig. S9.** Overexpression of DSG3 suppresses FAK and p‐FAK.

**Fig. S10.** Modulation of DSG3 expression has an impact on actin stress fibres.

**Table S1.** Clinical characteristics of oral cell lines.
**Table S2.** Analysis of the gene‐disease association and the number of publications (data retrieved from MalaCard).
**Table S3.** RT‐qPCR primers used in this study.

**Video S1.** Random cell migration of scrambled control siRNA treated H157 cells seeded sparsely. After two days, cells were treated with MMC at the concentration of 3 µg/ml for 3 hours before time‐lapse microscopy for 24 hours at an interval of 10 minutes. The video was ten frames per second.

**Video S2.** Random cell migration of YAP siRNA‐1 treated H157 cells seeded sparsely. After two days, cells were treated with MMC at the concentration of 3 µg/ml for 3 hours before time‐lapse microscopy for 24 hours at an interval of 10 minutes. The video was ten frames per second.

**Video S3.** Random cell migration of scrambled control siRNA treated H413 cells seeded sparsely. After two days, cells were treated with MMC at the concentration of 3 µg/ml for 3 hours before time‐lapse microscopy for 24 hours at an interval of 10 minutes. The video was ten frames per second.

**Video S4.** Random cell migration of YAP siRNA‐1 treated H413 cells seeded sparsely. After two days, cells were treated with MMC at the concentration of 3 µg/ml for 3 hours before time‐lapse microscopy for 24 hours at an interval of 10 minutes. The video was ten frames per second.

**Video S5.** The scratch wounding assay in the H413‐Vect Ct line. Cells were seeded at confluent density overnight before being treated with MMC at the concentration of 10 µg/ml for 3 hours, then scratched and subjected to time‐lapse microscopy 4 hours later, at an interval of 20 minutes for 15 hours. The video was five frames per second.

**Video S6.** The scratch wounding assay of the H413‐hDsg3.myc line treated alongside the Vect Ct cells shown in Video S5. The video was made at five frames per second from time‐lapse microscopy at an interval of 20 minutes for 15 hours.

**Video S7.** Random cell migration of H413‐Vect Ct cells. Cells were seeded sparsely overnight before being treated with MMC at the concentration of 3 µg/ml for 3 hours and then subjected to time‐lapse microscopy at an interval of 20 minutes for 15 hours. The video was eight frames per second.

**Video S8.** Random cell migration of H413‐hDsg3.myc cells. Cells were seeded along with the Vect Ct cells shown in Video S7. The video was eight frames per second.

## Data Availability

The data that support the findings of this study are available in the supplementary material of this article, including Figs [Link], [Link], [Link], [Link], [Link], [Link], [Link], [Link], [Link], [Link], Videos [Link], [Link], [Link], [Link], [Link], [Link], [Link], [Link] and Table S1–S3.
