# Supplementary figures and images for: Desmoglein‐3 induces YAP phosphorylation and inactivation during collective migration of oral carcinoma cells
Source: Mol Oncol. 2022 Mar 1;16(8):1625–49. doi: 10.1002/1878-0261.13177 (PMC9019900; doi:10.1002/1878-0261.13177)

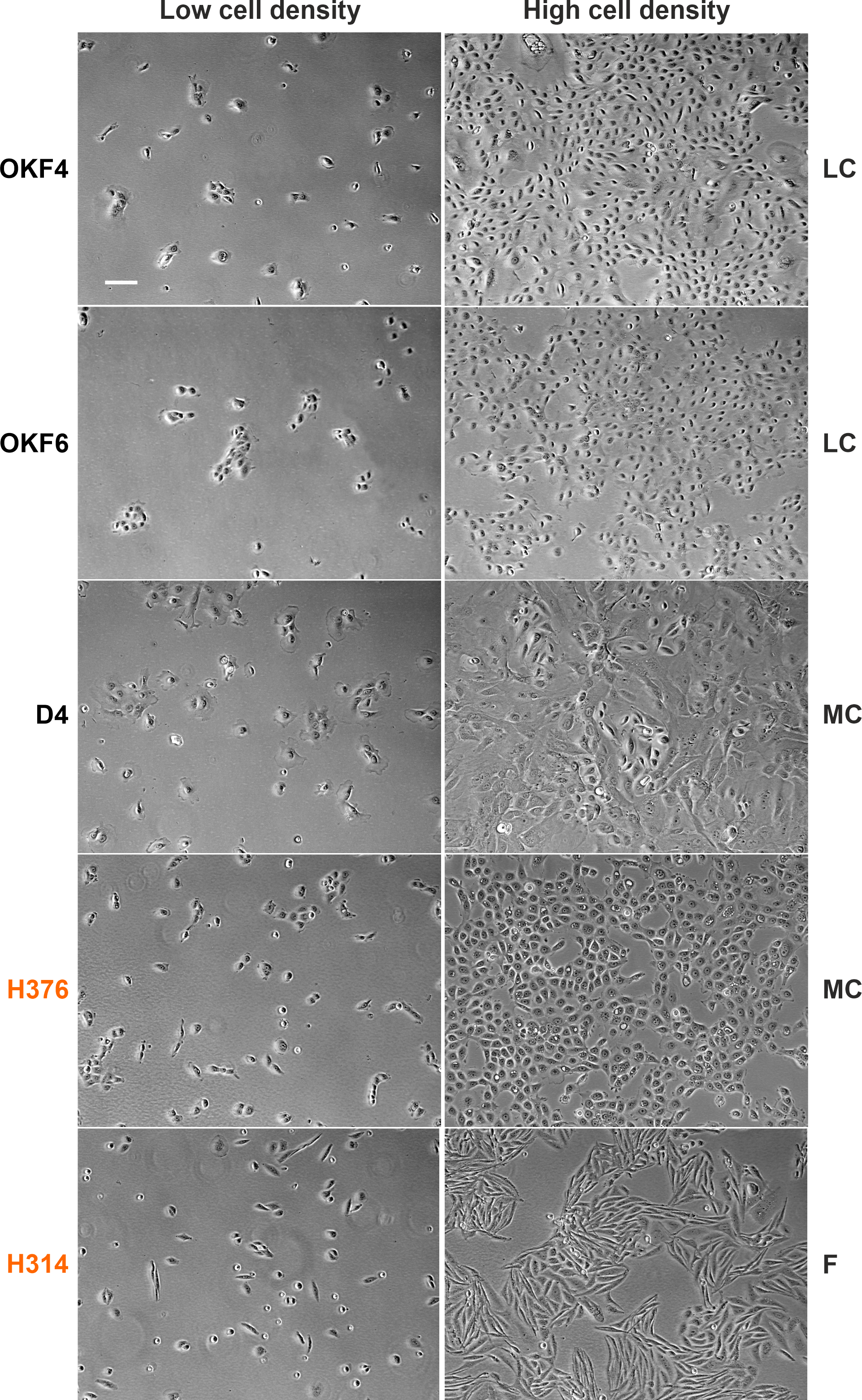

Supplement: Supplementary file 1 — Fig. S1. Phase‐contrast images of five oral floor of mouth keratinocyte cell lines. [file MOL2-16-1625-s019.tif]

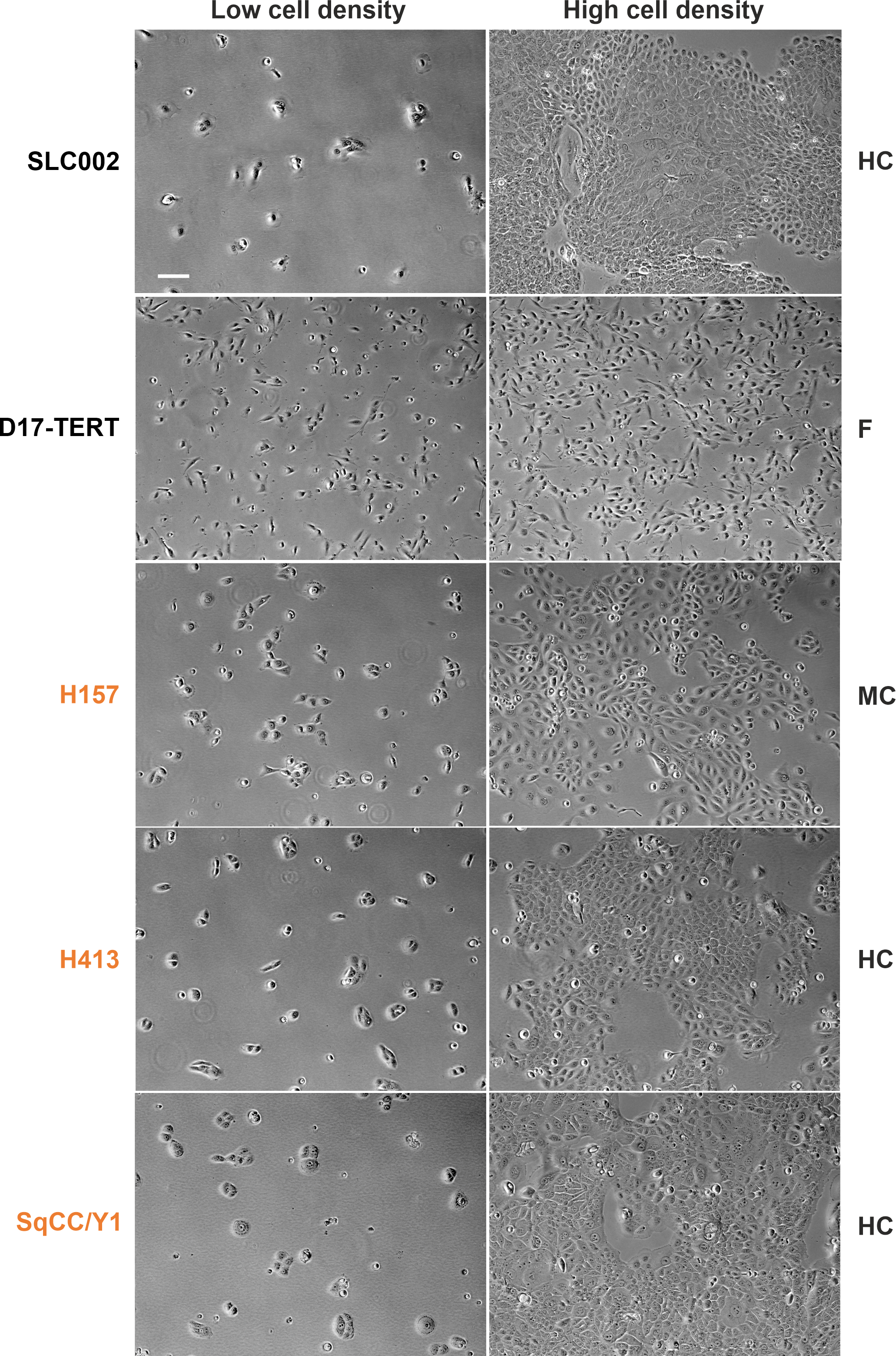

Supplement: Supplementary file 2 — Fig. S2. Phase‐contrast images of five oral buccal keratinocyte cell lines. [file MOL2-16-1625-s004.tif]

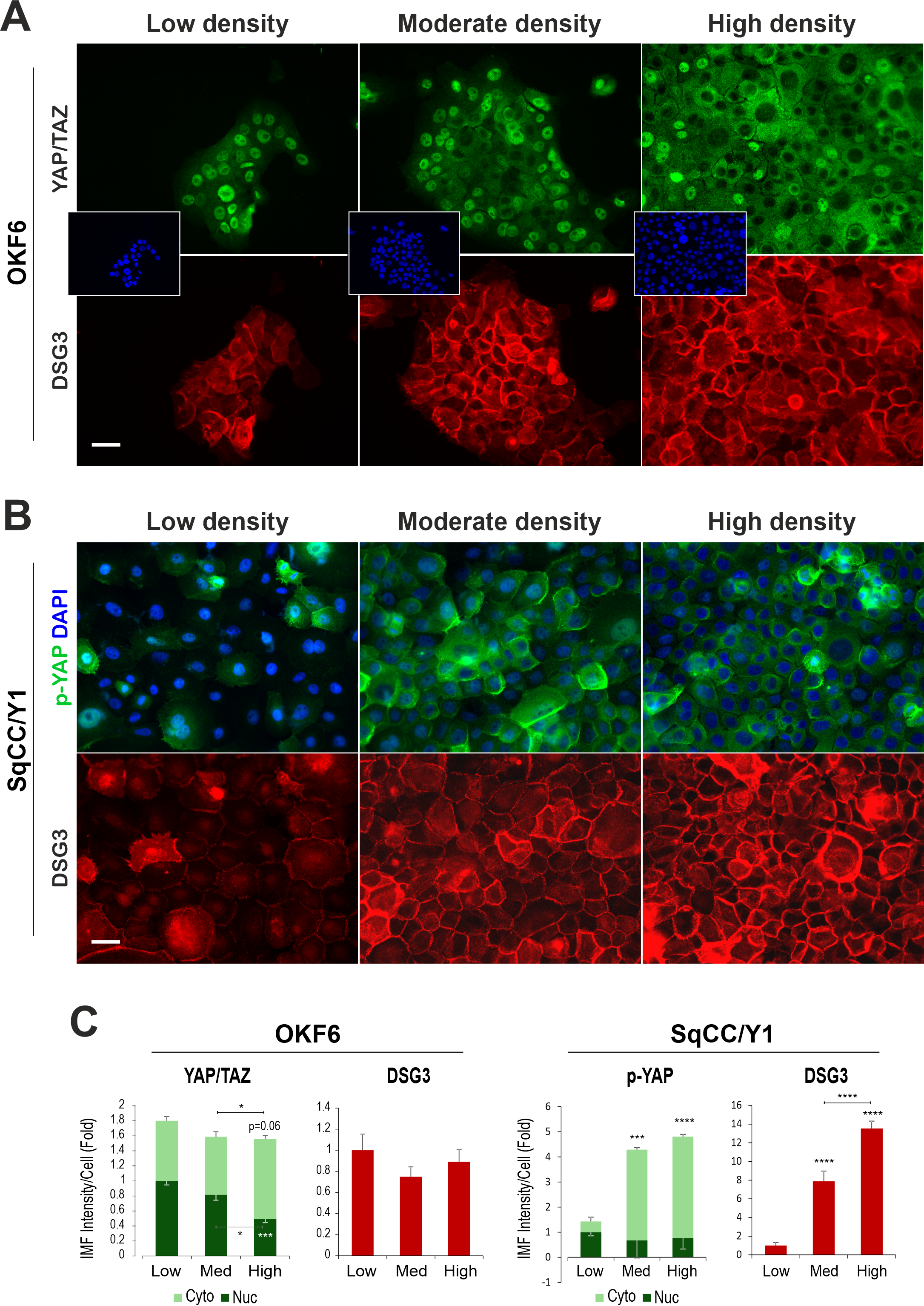

Supplement: Supplementary file 3 — Fig. S3. YAP exhibits cell density‐dependent subcellular translocation from the nucleus to the cytoplasm with concomitant elevated p‐YAP expression. [file MOL2-16-1625-s003.tif]

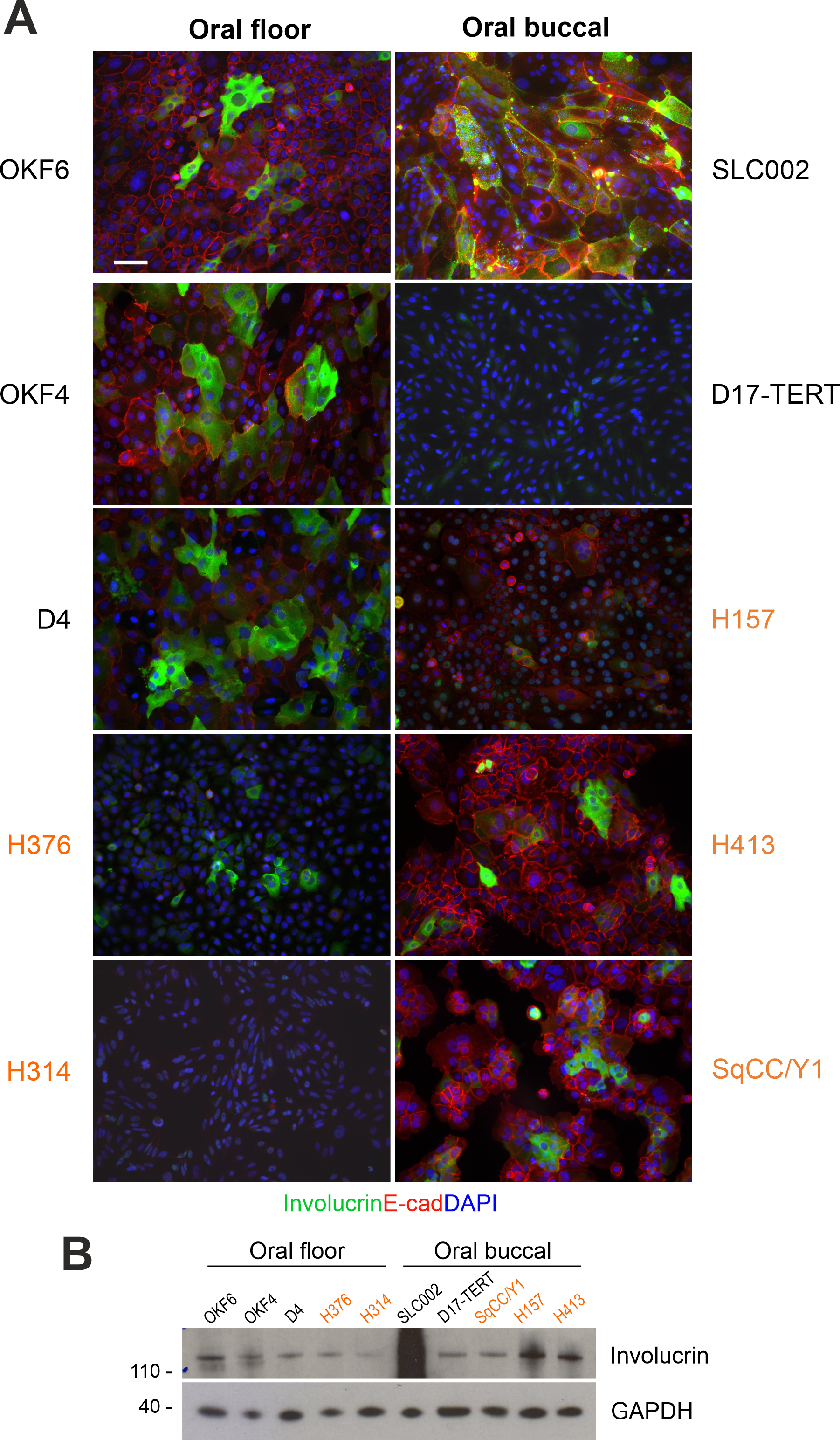

Supplement: Supplementary file 4 — Fig. S4. The differentiation marker Involucrin staining shows reduced or loss in oral dysplasia and carcinoma cell lines. [file MOL2-16-1625-s010.tif]

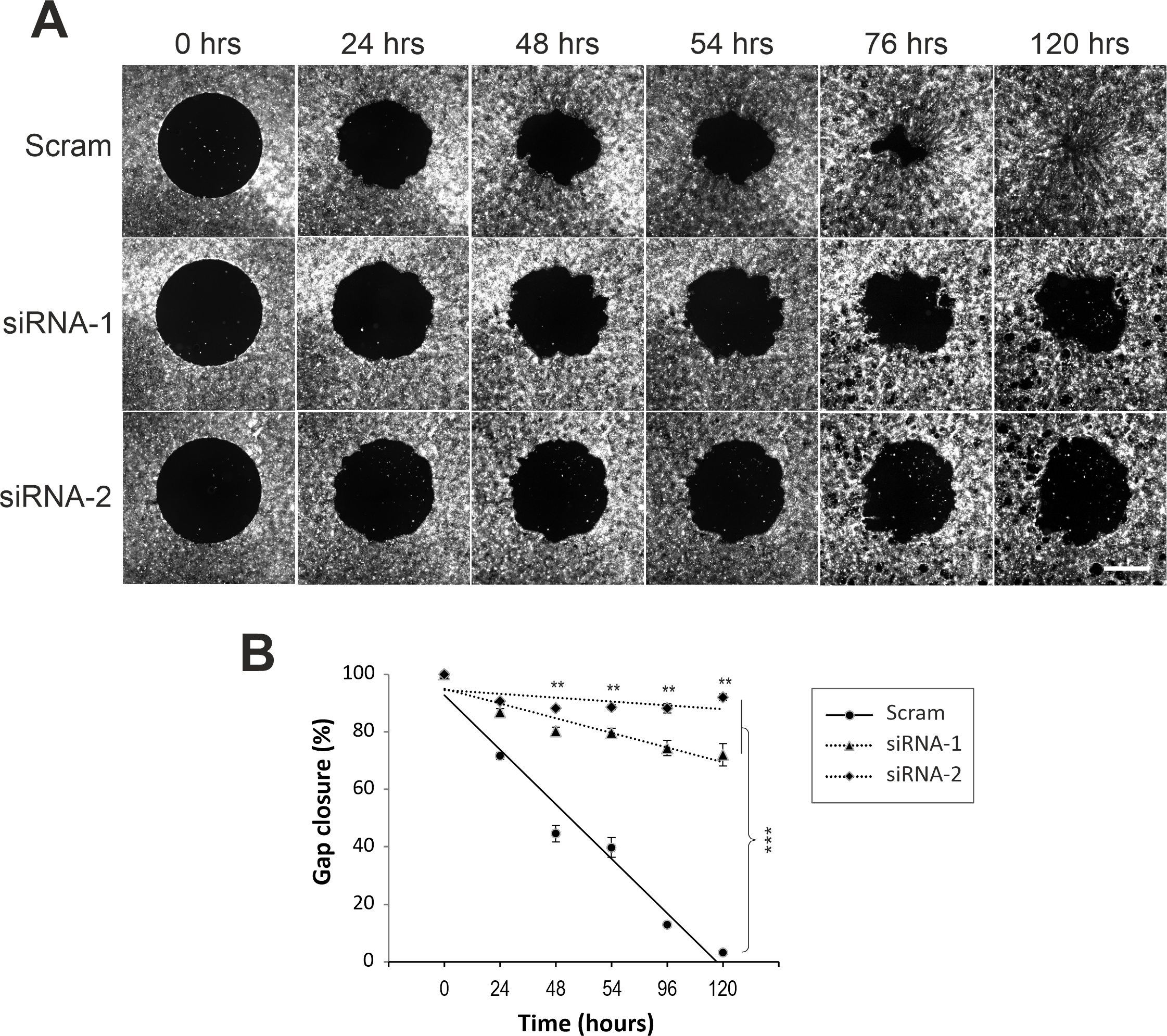

Supplement: Supplementary file 5 — Fig. S5. A time‐course study of Oris™ migration assay in H157 cell line. [file MOL2-16-1625-s016.tif]

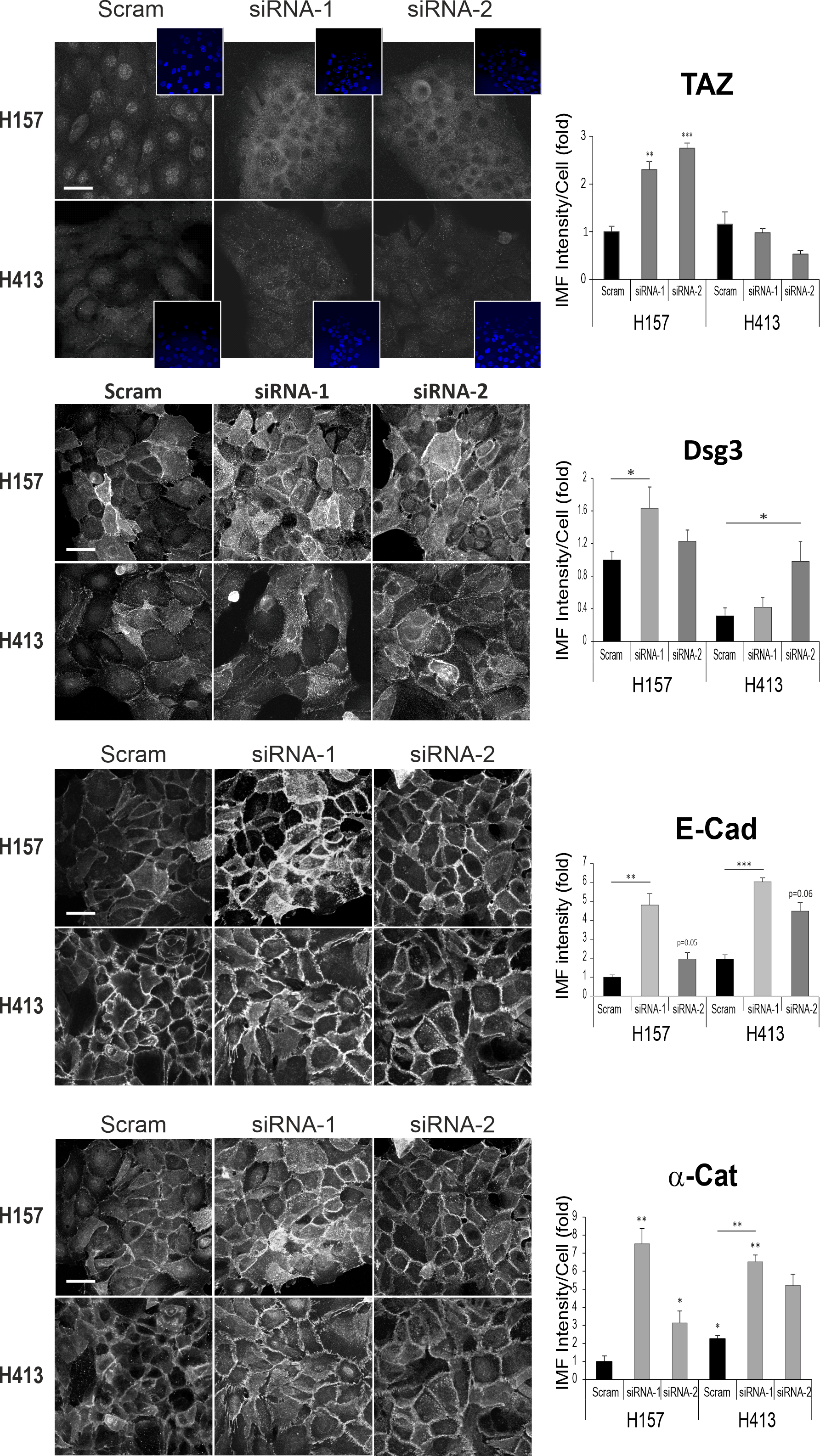

Supplement: Supplementary file 6 — Fig. S6. YAP knockdown causes increased cytoplasmic localisation of TAZ in the H157 line and enhanced expression of DSG3, E‐cadherin and α‐Catenin in both lines. [file MOL2-16-1625-s007.tif]

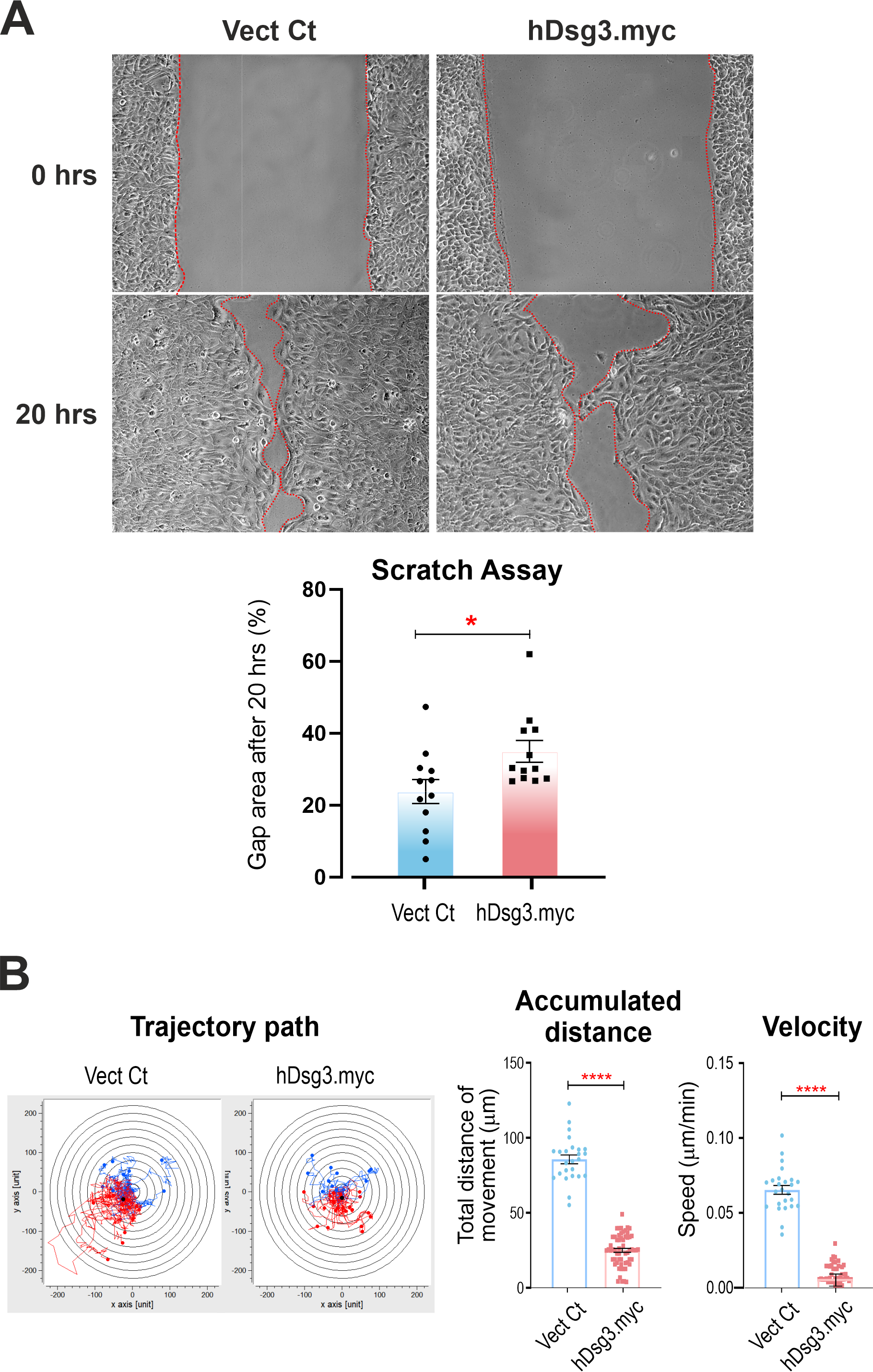

Supplement: Supplementary file 7 — Fig. S7. Overexpression of DSG3 inhibits both the collective and random cell migration in the OSCC H413 cell line. [file MOL2-16-1625-s001.tif]

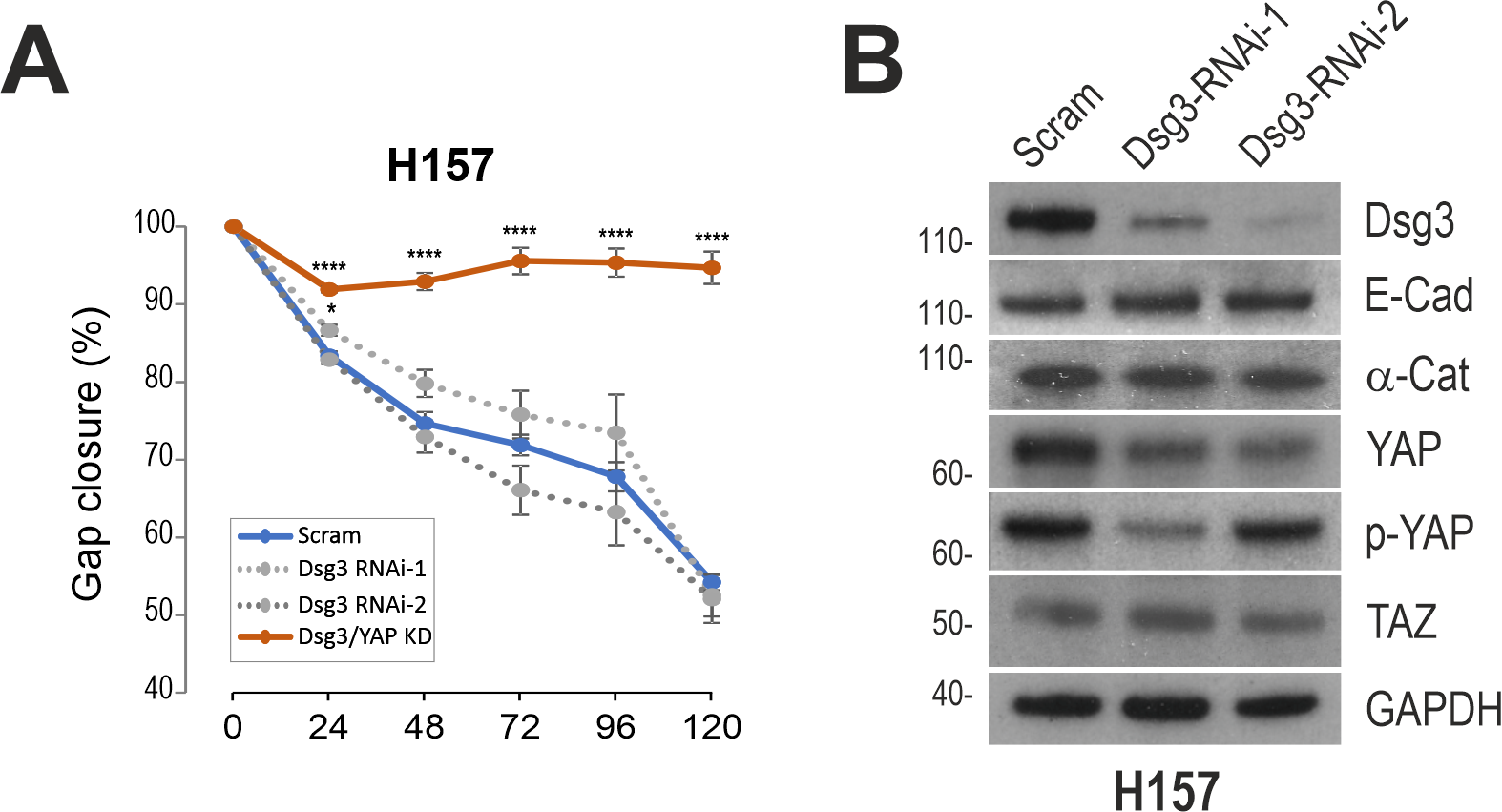

Supplement: Supplementary file 8 — Fig. S8. DSG3 knockdown causes a reduction of YAP and p‐YAP but with no evident effect on collective cell migration in OSCC cells. [file MOL2-16-1625-s017.tif]

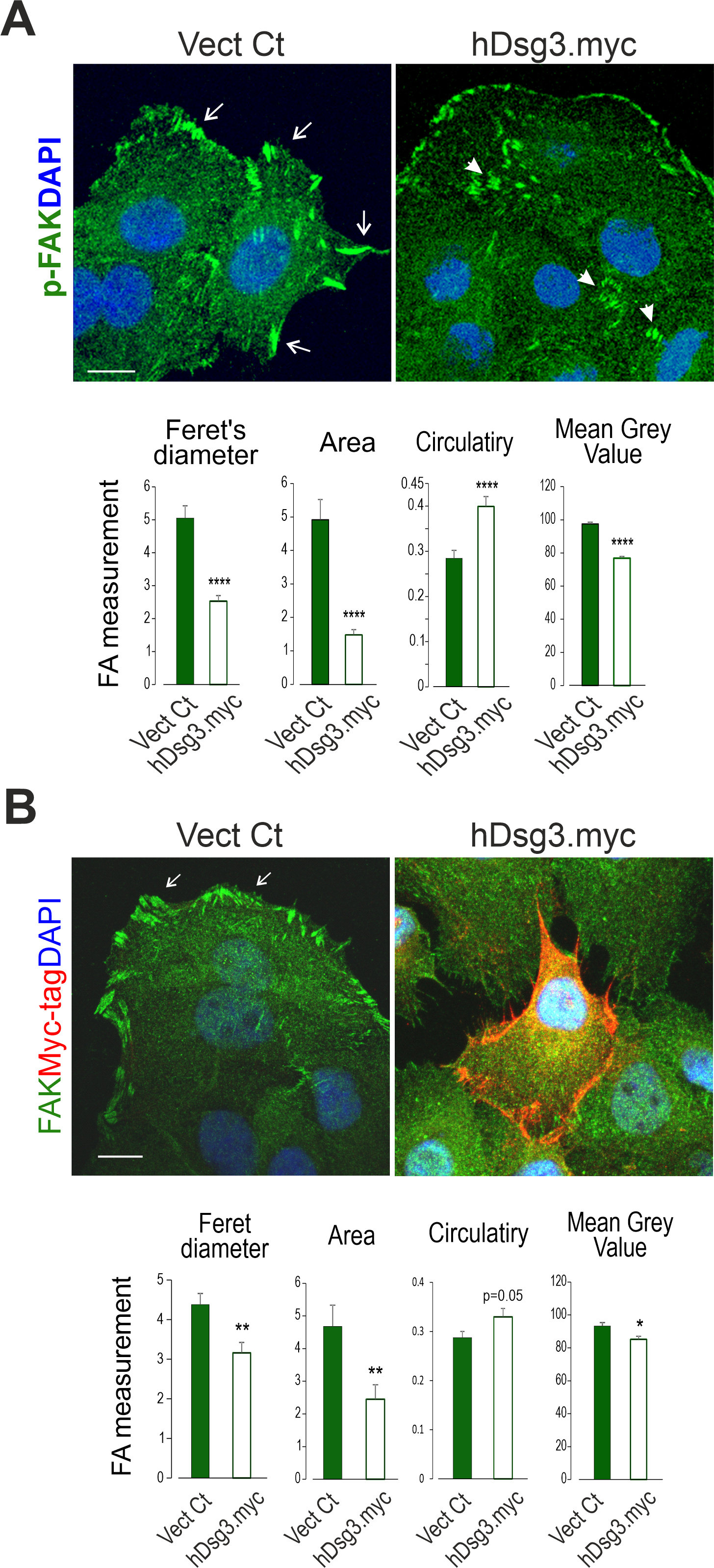

Supplement: Supplementary file 9 — Fig. S9. Overexpression of DSG3 suppresses FAK and p‐FAK. [file MOL2-16-1625-s008.tif]

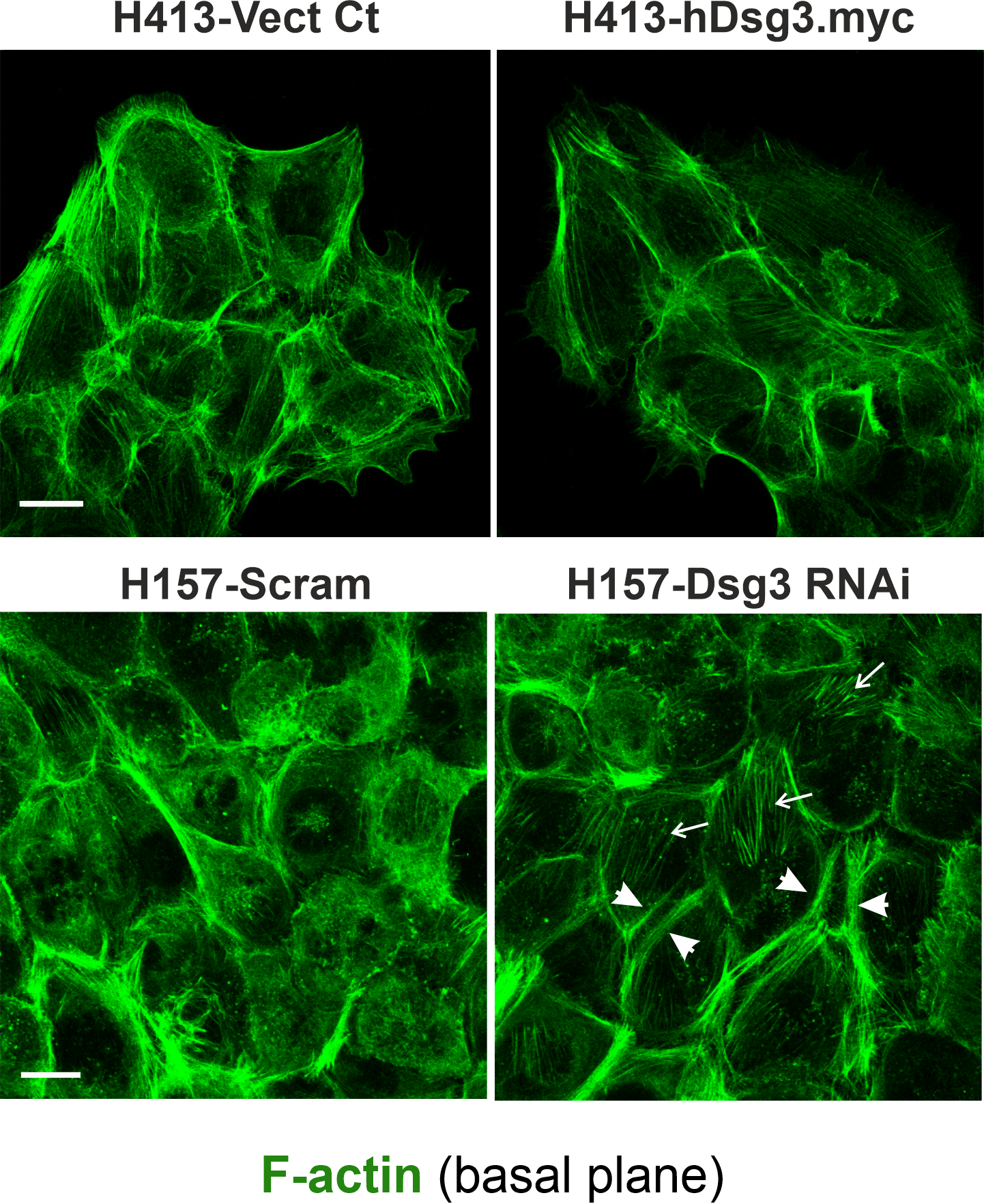

Supplement: Supplementary file 10 — Fig. S10. Modulation of DSG3 expression has an impact on actin stress fibres. [file MOL2-16-1625-s013.tif]
