# Supplementary material for: Desmoglein‐3 induces YAP phosphorylation and inactivation during collective migration of oral carcinoma cells
Source: Mol Oncol. 2022 Mar 1;16(8):1625–49. doi: 10.1002/1878-0261.13177 (PMC9019900; doi:10.1002/1878-0261.13177)
Supplement: Supplementary file 11 — Table S1. Clinical characteristics of oral cell lines. Table S2. Analysis of the gene‐disease association and the number of publications (data retrieved from MalaCard). Table S3. RT‐qPCR primers used in this study. [file MOL2-16-1625-s011.docx]

Ahmad et al., Supplementary Materials

**Supplemental Figures**

**Figure S1. Phase-contrast images of five oral floor of mouth keratinocyte cell lines.** Cells were grown in sparse or ~70% confluence in 100 mm culture dishes. Phase-contrast images were acquired with a 10x objective. Four categories in terms of morphology and compact of cells were defined, named highly compact (HC); medium compact (MC); loosely associated/compact (LC); and fibroblast-like, spindle-shaped with few attachments (F). HC-like morphology was not observed in this group of cell lines. Scale bars, 50 µm.

**Figure S2. Phase-contrast images of five oral buccal keratinocyte cell lines.** Cells were grown in sparse or ~70% confluence in 100 mm cell culture dishes and images were obtained with a 10x objective. Again, four categories as described in Figure S1 were used to define cellular morphology and are indicated on the right, *i.e.* HC: high compact; LC: loosely associated/compact; F: fibroblastic-like morphology; MC: medium compact. No LC trait was observed in this group of cell lines. Scale bars, 50 µm.

**Figure S3. YAP exhibits cell density-dependent subcellular translocation from the nucleus to the cytoplasm with concomitant elevated p-YAP expression.** **(A)** OKF6 cells grown at low, moderate and high confluence were double-stained with anti-YAP/TAZ and DSG3 antibodies. Increased nuclear exclusion with concomitantly cytoplasmic translocation of YAP/TAZ was evident in cells from low to high densities. **(B)** SqCC/Y1 cells plated at three different densities were labelled with anti-p-YAP and DSG3 antibodies. It showed a marked cell density-dependent increase of cytoplasmic p-YAP along with elevated DSG3 expression. Note that the membrane distribution of p-YAP was evident in some cells in the population of moderate density. **(C)** Quantitation of the images shown in A and B (n=4~5 fields/coverslip, *p<0.05, ***p<0.001, ****p<0.0001 determined by one-way ANOVA).

**Figure S4. The differentiation marker Involucrin staining shows reduced or loss in oral dysplasia and carcinoma cell lines.** **(A)** Immunostaining of Involucrin (green) and E-cadherin (red) in ten cell lines grown at approximately 70% confluence. Reduced expression of both proteins was evident in dysplasia and OSCC cell lines. **(B)** Western blotting showed the same trend as immunofluorescence with the loss of Involucrin in both dysplasia and OSCC lines. GAPDH was used as the loading control. Scale bar, 20 µm.

**Figure S5. A time-course study of Oris^TM^ migration assay in H157 cell line.** Cells were transfected with two YAP siRNAs or scrambled control siRNA for one day before being harvested and seeded in Oris^TM^ 96-well plate. The ability of cells to migrate into the detection zone was monitored for up to 5 days. The representative fluorescent images in each condition were shown in **(A)** and quantitation is shown in **(B)** (Mean ± SEM, **p<0.01, ***p<0.001 determined by one-way ANOVA).

**Figure S6. YAP knockdown causes increased cytoplasmic localisation of TAZ in the H157 line and enhanced expression of DSG3, E-cadherin and α-Catenin in both lines.** Confocal microscopy and image quantitation for the indicated proteins in H157 and H413 cell lines with and without YAP knockdown, respectively. Cells were transfected with two YAP siRNAs or scrambled control siRNA for one day before being harvested and plated on coverslips at approximately 70-80% confluence. Cells were fixed two days after siRNA transfection and immunostained for the indicated proteins. The DAPI channels for TAZ staining are shown in the inserts. Note that increased TAZ cytoplasmic distribution was detected only in H157 cells with YAP knockdown, with little effect in H413. DSG3 was also expressed at a higher level with richer membrane distribution in H157 than H413. YAP depletion caused elevated expression and membrane localisation of DSG3 in both cell lines. A similar effect was detected for E-cadherin and α-Catenin though both AJ proteins presented higher expression levels in H413 than H157. Double staining for E-cadherin (mouse Ab HECD-1) and α-Catenin (rabbit Ab to α-Catenin) was performed in both lines and it occurred that the images in the same fields were selected in different conditions for H413, respectively. (n=5 fields/sample, Mean ± SEM, *p<0.05, **p<0.01, ***p<0.001 determined by the Student’s *t*-test).

**Figure S7. Overexpression of DSG3 inhibits both the collective and random cell migration in the OSCC H413 cell line. (A)** The scratch wounding assay with representative images at 0- and 20-hours’ time points are shown on the top and the quantitation data displayed underneath (n=12, pooled from two independent experiments, Mean ± SEM, *p<0.05 determined by the Student’s *t*-test). Cells were plated at confluent density in a 6-well plate for one day before being treated with MMC and then the execution of this assay. The same experiment was also analysed by time-lapse microscopy (Video-5 and Video-6). **(B)** Random cell migration in Vect Ct and hDsg3.myc cell lines (Video-7 and Video-8). Cells were seeded sparsely in a 6-well place overnight before the time-lapse microscopy at 20 minutes intervals for 15 hours. Cell migration in five regions was monitored per condition. Manual cell tracking was performed with ImageJ. The trajectory path, accumulated distance and velocity of cell movement were shown (n=25~60 cells, **p<0.01, ****p<0.0001 determined by two-tailed Student’s *t*-test).

**Figure S8. DSG3 knockdown causes a reduction of YAP and p-YAP but with no evident effect on collective cell migration in OSCC cells**. **(A)** The graph shows the results of Oris^TM^ migration assay in H157 cells with DSG3 single gene knockdown (two hits) and DSG3/YAP double knockdown alongside scrambled control siRNA treated cells. The ability of the cells to migrate were monitored for up to 5 days (Mean ± SEM, Student’s *t*-test, ****p<0.0001). Note that DSG3 depletion showed no apparent effect on cell migration except for the cells with double YAP/DSG3 knockdown. **(B)** Western blotting analysis of the indicated proteins showed that DSG3 knockdown also caused a reduction of YAP and p-YAP.

**Figure S9. Overexpression of DSG3 suppresses FAK and p-FAK.** Fluorescent confocal image and morphological analysis of p-FAK and total FAK-associated FA in H413-Vect Ct and hDsg3.myc stable lines including all the parameters analysed in Figure 4F. A significant reduction of FA in both protein staining was shown in hDsg3.myc cells compared to the matched Vect Ct line. Arrows indicate different FA streaks in the two lines and arrowheads indicate enhanced p-FAK between cells in hDsg3.myc line. (n=9, Mean ± SEM, *p<0.05, **p<0.01, ***p<0.001, ****p<0.0001 determined by the Student’s *t-*test).

**Figure S10. Modulation of DSG3 expression has an impact on actin stress fibres**. Confocal images of F-actin staining with A488 conjugated phalloidin showed that overexpression of DSG3 in H413-hDsg3.myc cells caused a decrease of F-actin staining signals and stress fibres compared to H413-Vect Ct cells. In contrast, DSG3 knockdown in H157 cells enhanced the stress fibre formation (arrows) coupled with the thick actin bundles located on either side of intercellular junctions (arrowheads). Images were focused on the basolateral planes of the cells. Scale bars, 10 µm.

**Supplementary Videos**

Video S1. Random cell migration of scrambled control siRNA treated H157 cells seeded sparsely. After two days, cells were treated with MMC at the concentration of 3 µg/ml for 3 hours before time-lapse microscopy for 24 hours at an interval of 10 minutes. The video was ten frames per second.

Video S2. Random cell migration of YAP siRNA-1 treated H157 cells seeded sparsely. After two days, cells were treated with MMC at the concentration of 3 µg/ml for 3 hours before time-lapse microscopy for 24 hours at an interval of 10 minutes. The video was ten frames per second.

Video S3. Random cell migration of scrambled control siRNA treated H413 cells seeded sparsely. After two days, cells were treated with MMC at the concentration of 3 µg/ml for 3 hours before time-lapse microscopy for 24 hours at an interval of 10 minutes. The video was ten frames per second.

Video S4. Random cell migration of YAP siRNA-1 treated H413 cells seeded sparsely. After two days, cells were treated with MMC at the concentration of 3 µg/ml for 3 hours before time-lapse microscopy for 24 hours at an interval of 10 minutes. The video was ten frames per second.

Video S5. The scratch wounding assay in the H413-Vect Ct line. Cells were seeded at confluent density overnight before being treated with MMC at the concentration of 10 µg/ml for 3 hours, then scratched and subjected to time-lapse microscopy 4 hours later, at an interval of 20 minutes for 15 hours. The video was five frames per second.

Video S6. The scratch wounding assay of the H413-hDsg3.myc line treated alongside the Vect Ct cells shown in Video S5. The video was made at five frames per second from time-lapse microscopy at an interval of 20 minutes for 15 hours.

Video S7. Random cell migration of H413-Vect Ct cells that were seeded sparsely overnight before being treated with MMC at the concentration of 3 µg/ml for 3 hours and then subjected to time-lapse microscopy at an interval of 20 minutes for 15 hours. The video was eight frames per second.

Video S8. Random cell migration of H413-hDsg3.myc cells seeded along with the Vect Ct cells shown in Video S7. The video was eight frames per second.

**Supplementary Table S1. Clinical characteristics of oral cell lines**

| **Cell Line** | **Sex** | **Age** | **Site** | **Lesion** | **Tumour Stage** | **Lymph Node Metastasis** | **Differentiation** | **p53 status** | **P16 INK4A status** |
| --- | --- | --- | --- | --- | --- | --- | --- | --- | --- |
| OKF4 | M | 28 | FOM | N/A | N/A | N/A | Normal | Normal | Loss |
| OKF6 | M | 57 | FOM | N/A | N/A | N/A | Normal | Normal | Loss |
| D4 | M | 51 | FOM | Leukoplakia | T_is_ | - | Dysplastic | Heterozygous | Loss |
| H376 | F | 40 | FOM | Carcinoma | T_3_ | + | Well | Nonsense | Loss |
| H314 | M | 82 | FOM | Carcinoma | T_2_ | + | Moderately | Missense | Loss |
| SLC002 | NK | NK | BM | N/A | N/A | N/A | Normal | Normal | Normal |
| D17-TERT | M | 61 | BM | Leukoplakia | T_is_ | - | Dysplastic | WT | Loss |
| SqCC/Y1 | NK | NK | BM | Carcinoma | NK | NK | Well | Mutant | NK |
| H157 | M | 84 | BM | Carcinoma | T_2_ | + | Well | Nonsense | Loss |
| H413 | F | 53 | BM | Carcinoma | T_2_ | - | Moderately | Missense | Loss |

Abbreviations: M: male; F: female; BM: buccal mucosa; FOM: floor of mouth; NK: not known; WT: wild type; N/A: not applicable. Tumour stage was calculated using the STNMP staging system [33].

**Supplementary Table S2. Analysis of the gene-disease association and the number of publications (data retrieved from MalaCard)**

| **Gene** | **Gene-Disease Association** | **Number of Publication** | **Total Number of Publication** |
| --- | --- | --- | --- |
|  |  |  |  |
| *YAP1* | OSCC | 14 | 16 |
|  | OC | 2 |  |
| *PKP1* | OSCC | 1 | 2 |
|  | HNSCC | 1 |  |
| *DSC3* | OSCC | 1 | 1 |
| *SNAI1* | OSCC | 23 | 32 |
|  | HNSCC | 1 |  |
|  | OC | 8 |  |
| *TWIST1* | OSCC | 4 | 6 |
|  | HNSCC | 1 |  |
|  | OC | 1 |  |
| *CTGF* | OSCC | 2 | 5 |
|  | HNSCC | 1 |  |
|  | OC | 2 |  |
| *CYR61* | OSCC | 2 | 4 |
|  | HNSCC | 1 |  |
|  | OC | 1 |  |
| *TBX5* | OC | 1 | 1 |
| *DSG1* | OSCC | 1 | 1 |
| *BIRC5* | OSCC | 50 | 81 |
|  | HNSCC | 8 |  |
|  | OC | 23 |  |
| *CDH2* | OSCC | 8 | 10 |
|  | OC | 2 |  |
| *DSP* | OC | 1 | 1 |
| *DSG3* | OSCC | 3 | 5 |
|  | OC | 2 |  |

Abbreviations: oral squamous cell carcinoma (OSCC); head and neck squamous cell carcinoma (HNSCC); oral cancer (OC).

| **Supplementary Table S3. RT-qPCR primers used in this study** | | |
| --- | --- | --- |
| **Gene** | **Product Size (bp)** | **Primers** |
| *B2M* (Control)* | 70 | Forward 5’-AAGTGGGATCGAGACATGTAAG-3’ |
|  |  | Reverse 5’-GGAATTCATCCAATCCAAATGCG-3’ |
| *POLR2A* (Control)* | 73 | Forward 5’-GCAAATTCACCAAGAGAGACG-3’ |
|  |  | Reverse 5’-CACGTCGACAGGAACATCAG-3’ |
| *BIRC5** | 104 | Forward 5’-AGAACTGGCCCTTCTTGGA-3’ |
|  |  | Reverse 5’-ACACTGGGCCAAGTCTGG-3’ |
| *CCNA2** | 108 | Forward 5’-CCATACCTCAAGTATTTGCCATC-3’ |
|  |  | Reverse 5’-TCCAGTCTTTCGTATTAATGATTCAG-3’ |
| *CCNB1** | 102 | Forward 5’-CATGGTGCACTTTCCTCCTT-3’ |
|  |  | Reverse 5’-AGGTAATGTTGTAGAGTTGGTGTCC-3’ |
| *CDH1** | 96 | Forward 5’-GCCAGACACATTTATGGAACAG-3’ |
|  |  | Reverse 5’-GTGGAAATGGCACCAGTGT-3’ |
| *CDH2* | 131 | Forward 5’-CAGAATCAGTGGCGGAGATC-3’ |
|  |  | Reverse 5’-CAGCAACAGTAAGGACAAACATC-3’ |
| *CENPA** | 104 | Forward 5’-GCACCCAGTGTTTCTGTCAGT-3’ |
|  |  | Reverse 5’-CCAGACAGCATCGCAGAAT-3’ |
| *CMYC** | 138 | Forward 5’-CACCAGCAGCGACTCTGA-3’ |
|  |  | Reverse 5’-CTGTGAGGAGGTTTGCTGTG-3’ |
| *CTGF** | 249 | Forward 5’-CGACTGGAAGACACGTTTGG-3’ |
|  |  | Reverse 5’-AGGCTTGGAGATTTTGGGAG-3’ |
| *CTNNA1** | 130 | Forward 5’-GCCTACCTGCAACGCATC-3’ |
|  |  | Reverse 5’-CCTGGATCAGGGACATGG-3’ |
| *CTNNB1** | 137 | Forward 5’-TTGGATATCGCCAGGATGAT-3’ |
|  |  | Reverse 5’-CCCATCAACTGGATAGTCAGC-3’ |
| *CYR61** | 190 | Forward 5’-GAGTGGGTGTGTGACGAGGAT-3’ |
|  |  | Reverse 5’-GGTTGTATAGGATGCGAGGCT-3’ |
| *DSC1** | 252 | Forward 5’-CGAGCTCACAAACTCCTACA-3’ |
|  |  | Reverse 5’-CAACACACTGGCGACTTGAG-3’ |
| *DSC2** | 207 | Forward 5’-CCACTTGCCAACATTTACTCG-3’ |
|  |  | Reverse 5’-GCCAAAACCAATGAAGGAGT-3’ |
| *DSC3** | 207 | Forward 5’-GTACAAGACATGGATGGCCA-3’ |
|  |  | Reverse 5’-CACTGCCAATTGGAGAGTCA-3’ |
| *DSG1** | 233 | Forward 5’-GGCCAGTATGCTCTTGCTGT-3’ |
|  |  | Reverse 5’-CAGCTAACTGGATGGCAGTA-3’ |
| *DSG2** | 211 | Forward 5’-GAAGCAAGAGATGGCAATGG-3’ |
|  |  | Reverse 5’-GGTTCTGATAATTGGCTGGC-3’ |
| *DSG3** | 164 | Forward 5’-CCGAATCTCTGGAGTGGGAA-3’ |
|  |  | Reverse 5’-GCCCAAGGACTAGATGTAGA-3’ |
| *DSP** | 221 | Forward 5’-GCAGTCTACTGAAGCATACC-3’ |
|  |  | Reverse 5’-CGTAACCCAGACTACAGAAG-3’ |
| *ERBB4* | 150 | Forward 5’-GAGCTGAAGAGGGTAAAAGTCC-3’ |
|  |  | Reverse 5’-CATGAACTCCACATTTGCCTTG-3’ |
| *FAT1* | 138 | Forward 5’-GTTGCCAAACCTCTTGATGC-3’ |
|  |  | Reverse 5’-TGTAGAAAACTGAGGACGATGG-3’ |
| *FOXM1** | 131 | Forward 5’-AAACCTGCAGCTAGGGATGTG-3’ |
|  |  | Reverse 5’-AGCCACTGGATGTTGGATAGG-3’ |
| *JUP** | 305 | Forward 5’-GGCCATTGTGCATCTCATCA-3’ |
|  |  | Reverse 5’-CTGCTCGCCATCTTCAAGTC-3’ |
| *LATS1* | 104 | Forward 5’-GATCCTCGACGAGAGCAGAT-3’ |
|  |  | Reverse 5’-TTCCAGCTCTGTTTGCGGTT-3’ |
| *LATS2* | 134 | Forward 5’-AACTCACAGATTTCGGCCTC-3’ |
|  |  | Reverse 5’-ACACCGACAGTTAGACACATC-3’ |
| *MOB1A* | 171 | Forward 5’-CCCTCTGAGGACCGAAGATGA-3’ |
|  |  | Reverse 5’-CCTCTCCCTCAGGCAACATA-3’ |
| *NF2* | 143 | Forward 5’-TGTGCTCCTGGCTTCTTACG-3’ |
|  |  | Reverse 5’-CCTCCCACATTTCCGGAGTC-3’ |
| *PKP1** | 249 | Forward 5’-CAGTTGATTGGGCTGAAGGA-3’ |
|  |  | Reverse 5’-CTACACTGTGAGGAACCTGA-3’ |
| *PKP2** | 238 | Forward 5’-GCTACTTTCATACAGCACGAGTG-3’ |
|  |  | Reverse 5’-GAAGCAAACCAGAGACTTGG-3’ |
| *PKP3** | 206 | Forward 5’-GCTCACAGACCTGGTGTTGA-3’ |
|  |  | Reverse 5’-ACCTCTATCAACCACGCCCT-3’ |
| *RUNX3* | 124 | Forward 5’-GTTCAACGACCTTCGCTTC-3’ |
|  |  | Reverse 5’-GTCCACGGTCACCTTGATG-3’ |
| *SAV1* | 114 | Forward 5’-AAGGCCCAATACAGGCATCC-3’ |
|  |  | Reverse 5’-TGCAGGTACCAGAAGGGACT-3’ |
| *SNAI1* | 150 | Forward 5’-CTAGGCCCTGGCTGCTACAA-3’ |
|  |  | Reverse 5’-CCTGGCACTGGTACTTCTTGA-3’ |
| *SNAI2* | 78 | Forward 5’-CTACAGCGAACTGGACACAC-3’ |
|  |  | Reverse 5’-TGTGGTATGACAGGCATGGAG-3’ |
| *STK3* | 95 | Forward 5’-GCAGAGAGCTACTGCAACACA-3’ |
|  |  | Reverse 5’-CCATAGCTTCTGTGATCAGGTCT-3’ |
| *STK4* | 164 | Forward 5’-GAGACGGTACAGCTGAGGAAC-3’ |
|  |  | Reverse 5’-ACAATCTGGCCGGTCTCTTT-3’ |
| *WWTR1* | 87 | Forward 5’-ATTCGAATGCGCCAAGAG-3’ |
|  |  | Reverse 5’-AACTGGGGCAAGAGTCTCAG-3’ |
| *TBX5* | 144 | Forward 5’-AGTGTGAGAATGGTGTTTCCG-3’ |
|  |  | Reverse 5’-TCTTATAGGGATGGTCTGTGGT-3’ |
| *TEAD1* | 139 | Forward 5’-ACGTCAAGCCTTTTGTGCAG-3’ |
|  |  | Reverse 5’-CGAAGCTTGGTTGTGCCAAT-3’ |
| *TEAD2* | 132 | Forward 5’-AACTGATCGCCCGCTACATC-3’ |
|  |  | Reverse 5’-TGGAAACCTGGTCCTTCAACT-3’ |
| *TEAD3* | 111 | Forward 5’-TGTACGGCCGAAATGAGTTGA-3’ |
|  |  | Reverse 5’-GCACCTTCTTCCGAGCTAGA-3’ |
| *TEAD4* | 77 | Forward 5’-CTCCACGAAGGTCTGCTCTT-3’ |
|  |  | Reverse 5’-GTCCATTCTCATAGCGAGCATA-3’ |
| *TOP2A** | 96 | Forward 5’-CAGTGAAGAAGACAGCAGCAAA-3’ |
|  |  | Reverse 5’-AAGCTGGATCCCTTTTAGTTCC-3’ |
| *TP73* | 147 | Forward 5’-GTTTACAAGAAAGCGGAGCAC-3’ |
|  |  | Reverse 5’-GTCATCCACATACTGCGAGAG-3’ |
| *TWIST1* | 153 | Forward 5’-TCAGCTACGCCTTCTCGGT-3’ |
|  |  | Reverse 5’-CCAGAGTCTCTAGACTGTCCAT-3’ |
| *YAP1** | 83 | Forward 5’-CCCAGATGAACGTCACAGC-3’ |
|  |  | Reverse 5’-GATTCTCTGGTTCATGGCTGA-3’ |
| *YWHAB* | 106 | Forward 5’-TGCAGTTACTTAGGGACAATCTC-3’ |
|  |  | Reverse 5’-CAGATCACAAAGCACGAGAAAC-3’ |
| *YWHAE* | 175 | Forward 5’-TAATTCCCCTGACCGTGCCT-3’ |
|  |  | Reverse 5’-ATTCTGCTCTTCACCGTCACC-3’ |
| *YWHAG* | 149 | Forward 5’-CGGCGAAGGCAACAATTAAG-3’ |
|  |  | Reverse 5’-GACAGGTCGTGGGTTTCTC-3’ |

Note: those marked with asterisks were used in our previous study [19] and the rest primers were designed in this study.
